# Pharmacokinetics, Tolerability, and Biomarker Profile of the Neurokinin 3 Receptor Antagonist Fezolinetant in Healthy Japanese Individuals: A 2‐Part, Randomized, Phase 1 Study

**DOI:** 10.1002/cpdd.1593

**Published:** 2025-09-12

**Authors:** Akira Koibuchi, Megumi Iwai, Kanji Komatsu, Kentaro Kuroishi, Mai Shibata, Masako Saito, Jace Nielsen, Jiayin Huang, Shunji Matsuki

**Affiliations:** ^1^ Astellas Pharma Inc. Chuo Tokyo Japan; ^2^ Astellas Pharma Global Development Northbrook IL USA; ^3^ Fukuoka Mirai Hospital Clinical Research Center Fukuoka Japan

**Keywords:** biomarkers, fezolinetant, Japanese, pharmacokinetics, safety

## Abstract

This 2‐part, randomized, placebo‐controlled, double‐blind, Phase 1 study analyzed the pharmacokinetics, safety, and biomarker profile of fezolinetant in healthy Japanese individuals. Part 1: male participants received single doses of placebo or fezolinetant 15 or 60 mg. Part 2: male and premenopausal and postmenopausal female participants received a single dose of placebo or fezolinetant 180 mg, followed by a 2‐day washout, and multiple dosing once daily for 10 days. Fezolinetant was rapidly absorbed with peak concentrations 1‐2 hours after single‐dose administration; plasma levels subsequently declined (half‐life range, 3.29‐7.24 hours). Only slight accumulation (area under the concentration–time curve accumulation ratio, 1.46‐1.57) was observed after once‐daily multiple‐dose administration. No serious/severe treatment‐emergent adverse events were observed; the only fezolinetant‐related treatment‐emergent adverse event was mild uterine bleeding in 1 premenopausal woman and 1 postmenopausal woman. Concentration‐QT analysis showed that fezolinetant does not have a clinically significant effect on QT interval. Fezolinetant produced dose‐dependent reductions in luteinizing hormone and slight reductions in follicle‐stimulating hormone; levels subsequently returned to baseline 48 hours or fewer after dosing. This analysis shows that fezolinetant doses up to 180 mg had an acceptable safety, pharmacokinetic, and biomarker profile. This study clarifies the safety, pharmacokinetic, and biomarker profiles of fezolinetant in Japanese individuals.

Individuals experiencing menopause commonly report vasomotor symptoms (VMS), which are characterized by hot flashes and night sweats.^1^, ^2^ These symptoms can have a substantial impact on quality of life, with individuals frequently identifying problems with sleep, mood, concentration, and depression.^3^, ^4^ Epidemiological studies have indicated that moderate to severe VMS are reported by approximately 16% of women from Japan who are aged 40‐65 years.^5^ Common menopausal symptoms that have been reported in this population include feeling tired or worn out, decreased physical strength, sweating, and decreased stamina.

The thermoregulatory center of the hypothalamus is innervated by kisspeptin‐neurokinin B‐dynorphin (KNDy) neurons, which are stimulated by the neuropeptide neurokinin B via neurokinin 3 receptor (NK3R) activation and inhibited by estrogen negative feedback.^6^ During menopause, declining estrogen levels leave NK3R‐mediated activation unopposed, resulting in overstimulation of KNDy neurons. The overstimulated thermoregulatory center becomes hypersensitive to external cues from peripheral sensors, activating heat dissipation effectors, resulting in sweating and vasodilation, which are experienced as VMS. The nonhormonal NK3R antagonist fezolinetant acts by blocking neurokinin B binding, which moderates KNDy neuronal activity, thereby reducing the frequency and severity of VMS due to menopause (see Fraser et al.^7^ for the chemical structure of fezolinetant).

Fezolinetant is primarily metabolized to form ES259564 by oxidation of the methyl group in the methylthiadiazole ring (Figure S1), which is mainly mediated by cytochrome P450 (CYP)1A2. In support of this finding, a recent Phase 1 study found that the strong CYP1A2 inhibitor fluvoxamine increased fezolinetant exposure about ninefold, while smoking, which induces CYP1A2, reduced fezolinetant exposure by approximately half.^8^ In terms of the metabolite, ES259564 is approximately 20‐fold less potent than fezolinetant and elimination occurs mainly via urine. Transporters are thought to have a limited influence on fezolinetant, as shown by the fact that fezolinetant is not a substrate of P‐glycoprotein, breast cancer resistance protein, or organic anion‐transporting polypeptide (OATP)1B1 or OATP1B3 (data on file). Conversely, ES259564 is a substrate of P‐glycoprotein but is not a substrate of breast cancer resistance protein, OATP1B1, OATP1B3, organic anion transporter 1, organic anion transporter 3, organic cation transporter 2, or multidrug and toxin extrusion 1 or 2‐K (data on file).

The efficacy and safety of fezolinetant have been demonstrated in several clinical studies, including the Phase 3 SKYLIGHT studies.^9^, ^10^, ^11^, ^12^, ^13^ The findings from these studies contributed to the approval of fezolinetant for the treatment of moderate to severe VMS in many regions, including North America, Europe, Asia, and Australia at a dose of 45 mg once daily. The SKYLIGHT studies were conducted across multiple locations in North America and Europe in a predominantly White population (80%).^11^, ^12^, ^13^

Race can influence the pharmacokinetics (PK) and biomarker profile of certain drugs.^14^, ^15^, ^16^ The frequencies of alleles in the *CYP1A2* gene, which encodes the enzyme of the main metabolic pathway for fezolinetant, differ between Japanese and White individuals.^17^ However, the clinical impacts of these genetic variations remain unconfirmed due to controversial findings.^18^, ^19^ Additionally, CYP1A2 activity is known to be affected by various environmental factors and have large interindividual variability.^16^, ^17^ Thus far, the PK and biomarker profiles of fezolinetant have been evaluated predominantly in White individuals. In the first‐in‐human study involving healthy men and premenopausal women from Belgium, single‐dose fezolinetant produced linear increases in exposure across a range of doses from 3 to 180 mg in men, and maximum concentrations (C_max_) were achieved after 1.5‐2.0 hours.^20^ This study also showed that fezolinetant produced decreases in luteinizing hormone (LH) concentrations, which consequently inhibited the daily rises in estradiol and progesterone in women and decreased testosterone levels in men, although little impact on follicle‐stimulating hormone (FSH) levels was noted. A Phase 2a study that enrolled predominantly White individuals (98.9%) also showed that decreases in plasma LH were obtained at peak fezolinetant levels, which occurred 3 hours after dosing.^9^ As fezolinetant studies mainly enrolled White individuals, there is a clinical need to study the PK and pharmacological properties of fezolinetant in individuals with ethnic backgrounds differing from other geographical regions, including in Asian populations.

The aim of this Phase 1 study was to evaluate the PK, safety, tolerability, and biomarker profile of fezolinetant in healthy Japanese individuals. We also planned to characterize the PK of ES259564, the major metabolite of fezolinetant. The PK results from this study helped inform the dosing regimen for the Japanese phase 2 STARLIGHT study, namely, fezolinetant 15 and 30 mg once daily.^21^ The STARLIGHT study showed that both doses can significantly reduce the frequency of hot flashes compared with placebo. In addition, the safety analyses from STARLIGHT showed that fezolinetant was well tolerated, and no safety signals of concern were noted for either dose.

## Methods

### Study Design

This Phase 1 healthy volunteer study involving Japanese individuals was conducted at Fukuoka Mirai Hospital, Medical Corporation SOUSEIKAI, Fukuoka, Japan (ClinicalTrials.gov: NCT03436849). The study was initiated (date of first informed consent) on February 22, 2018, and was completed (date of last evaluation) on May 23, 2018. The study protocol was approved by the Hakata Clinic Institutional Review Board, Fukuoka, Japan. The study was conducted according to principles consistent with the Declaration of Helsinki, Good Clinical Practice, International Committee on Harmonisation guidelines, and all applicable laws and regulations. All participants provided written informed consent before screening procedures were undertaken.

This was a Phase 1, 2‐part, randomized, placebo‐controlled, double‐blind study. Owing to the potential administration schedules and clinical indications for fezolinetant at the time this study was conducted, once‐daily doses of up to 180 mg were administered to various populations, including male participants and premenopausal and postmenopausal female participants.

Cohorts 1‐1, 1‐2, and 2‐1 comprised healthy Japanese participants who were assigned male at birth, aged 20 years or older, but younger than 45 years, with a body weight of 50 or greater to less than 80 kg and a body mass index (BMI) of 17.6 or greater to less than 26.4 kg/m^2^. Two of the Part 2 cohorts (2‐2a and 2‐2b) comprised healthy female participants who were assigned female at birth, aged 20 years or older, but younger than 65 years, with a body weight of 40 or greater to less than 70 kg, and a BMI of 17.6 or greater to less than 26.4 kg/m^2^. Premenopausal female participants were enrolled in Cohort 2‐2a and had to have regular menstrual cycles (25‐38 days) for 3 months before study drug initiation. Postmenopausal female participants, defined as those who had their last menses at least 1 year before screening and whose FSH level was above 30 mIU/mL at screening, were included in Cohort 2‐2b.

Individuals were excluded if they had a history of heart disease, hepatic or renal disorders, or hematologic or immune disorders or abnormal vital signs, laboratory results, or electrocardiogram (ECG) findings. Female participants were excluded if they had been pregnant in the 6 months or breastfeeding in the 3 months before screening. Additional exclusion criteria included use of any drug (including without a prescription) within 2 weeks of hospital admission, use of a drug or any therapy that affects sex hormones (including oral contraceptives, intrauterine system, or hormone replacement therapy) or metabolic enzymes within 3 months of admission, smoking 10 or more cigarettes/day, consumption of caffeine of 500 mg/day or greater or of alcohol 30 units/week or greater, or drug dependency/abuse. Individuals who had previously received fezolinetant or who were deemed unsuitable by the investigator were also excluded.

Part 1 was a single‐dose investigation that involved two cohorts who received placebo or fezolinetant 15 or 60 mg in a parallel manner. Healthy Japanese adult male participants were randomized (3:1) to receive single oral doses of fezolinetant or placebo during a 5‐day, 4‐night inpatient hospital stay. Cohort 1‐1 received fezolinetant 15 mg, and Cohort 1‐2 received 60 mg. Doses were administered as single capsules with 150 mL of water after a 10‐hour fast.

Part 2 was a single‐ and multiple‐dose investigation involving 3 cohorts consisting of healthy male participants (Cohort 2‐1), premenopausal female participants (Cohort 2‐2a), and postmenopausal female participants (Cohort 2‐2b). Cohorts 2‐2a and 2‐2b were conducted in a parallel manner. Participants were randomized (3:1) to receive single and, subsequently, multiple doses of fezolinetant 180 mg or placebo once daily during a 17‐day, 16‐night inpatient stay. The enrolled individuals initially received a single dose of study medication and then, after a 2‐day washout, received the same dose once daily for 10 days. The single dose was administered with 150 mL of water after a 10‐hour fast. Multiple‐dose treatment was administered with 150 mL of water after breakfast on Days 1 through 9 and after a 10‐hour fast on Day 10. For each dose, participants received two capsules of placebo or fezolinetant 90 mg. Premenopausal female participants (Cohort 2‐2a) began the inpatient stay during the first 3 days of their menstrual cycle.

In both parts, the randomization procedure was conducted by initially assigning participants to randomization numbers. The randomization number was given to the participants according to the order of informed consent. Allocation of the study drug to the randomization number was performed by the assignment manager or person in charge.

The participants were randomly allocated to fezolinetant or placebo using a method that ensured that the participants and related study‐site staff were unaware which drug was being administered. To maintain blinding of the participants and related staff, individuals who were enrolled in the same cohort were given the same number of capsules. During the study, the assignment managers, the bioanalytical laboratory, and the PK analysis managers were not blinded to the study drug allocation. Transition to the next part or cohort was determined under blinded conditions by a Safety and Pharmacokinetics Review Committee composed of principal investigators as well as medical and clinical pharmacology representatives from the sponsor.

The external appearances of the fezolinetant 15‐, 60‐, and 90‐mg capsules were the same as the external appearance of the placebo capsule. The assignment manager, or person in charge, confirmed the indistinguishability of the study drugs before preparation for dispensing and at the end of the study.

### Pharmacokinetics

Plasma samples for fezolinetant and ES259564 measurement and PK parameter analysis were collected before dosing and 1, 1.5, 2, 3, 4, 6, 8, 12, 16, 24, and 48 hours after the single doses and at the same assessment times before and after the last dose (Day 10) of the multiple‐dose period. Samples were also acquired before study drug administration on Days 2, 3, 5, and 7. Concentrations of fezolinetant and ES259564 were measured in the plasma samples using a validated liquid chromatography with tandem mass spectrometry method at Sumika Chemical Analysis Service, Ltd. (Osaka, Japan). Fezolinetant and ES259564 were isolated from plasma by solid phase extraction using an Oasis HLB 96‐well plate, 30 mg (Waters Corp.). A structural analog of fezolinetant (ES246567) was used as the internal standard for each analyte. The extracts were analyzed on a Kinetex C18 100A, 3.0 × 50 mm, 2.6 µm column (Phenomenex) using the gradient elution mode. The mobile phases used were methanol/water/trifluoroacetic acid (100:900:0.5, v/v/v, A) and methanol/water/trifluoroacetic acid (900:100:0.5, v/v/v, B). An API5000 (SCIEX) equipped with an atmospheric pressure chemical ionization interface was used to generate positive ions for mass spectrometric detection. The mass transitions (m/z) used were 359.2 → 123.0 for fezolinetant, 375.0 → 123.0 for ES259564, and 406.2 → 123.0 for ES246567.

The method was validated over a range of 1‐1000 ng/mL for each analyte. The intra‐ and interprecision (coefficient of variation) values were between 1.5% and 7.3% for fezolinetant, and between 1.1% and 8.2% for ES259564. The intra‐ and interassay accuracy values were ‐10.0% to 0.5% for fezolinetant and ‐6.3% to 5.6% for ES259564. The limit of quantitation for each analyte was 1 ng/mL in 0.05 mL of plasma.

### Safety and ECG Assessments

Safety was assessed using treatment‐emergent adverse events (TEAEs), vital signs, laboratory assessments (hematology, biochemistry, urinalysis), ECGs, body weight, and any menstrual cycle changes (premenopausal female participants only). TEAEs were collected from the time of informed consent until the end of the study.

Triplicate 12‐lead ECGs were digitally recorded after the participant had been in a supine position for 5 minutes with approximately 1‐min intervals between the recordings. Parameters were assessed for the ECGs that were conducted before dosing (23, 22, and 20 hours before dosing on Day 1) and 1, 2, 4, and 24 hours after dosing on Day 1 for Part 1 and at the same time points on Day 10 for Part 2. If blood sampling was designated at the same time, sampling was performed after ECG measurement. ECG intervals were assessed in a blinded manner by a central ECG laboratory (eResearchTechnology Inc.). Cardiac safety specialists reviewed all ECG data for the correct lead and beat selection using adjudicated calipers. A cardiologist interpreted all the ECG data generated in this study.

### Concentration–QTcF Assessments

Using the ECG results, the relationships between fezolinetant or ES259564 concentrations and QT intervals were explored. QT intervals were corrected for heart rate using Fridericia's formula (QTcF) and were analyzed in terms of the change from baseline in QTcF (dQTcF) results. The dQTcF intervals were calculated by subtracting the baseline result from each observation before dosing and at 1, 2, 4, and 24 hours after dosing on Days 1 and 10. Baseline was defined as the mean of the 3 ECG results at ‐23, ‐22, and ‐20 hours on Day ‐1. Participants who received placebo were included in the analysis and were assigned a zero value for plasma concentrations. Plasma concentrations that were below the limit of quantification were included in the analysis and set to 0. All data from both parts were pooled and subjected to the analysis.

### Biomarkers

Serum samples for the measurement of biomarkers were collected at 3 time points with at least 2‐hour intervals on Day −1 (mean of measurements at the 3 time points was used as baseline) and the same time points as the PK samples on Day 1. Outcomes included assessing LH, FSH, sex hormone–binding globulin (SHBG), total testosterone, and free testosterone levels in Part 1 and LH, FSH, SHBG, total testosterone, free testosterone, estradiol (in female participants), and progesterone (in female participants) levels in Part 2. LH, FSH, and estradiol in blood were measured at SRL, Inc. using a chemiluminescence immunoassay (Architect reagent, pretrigger, trigger, and 2000SR analyzer; Abbott Japan),^22^, ^23^ total testosterone (Elecsys Testosterone II, ProCell M, CleanCell M, diluent; Roche Diagnostics; and Modular Analytics System, Hitachi Ltd.) and progesterone (Elecsys Progesterone III, ProCell M, CleanCell M, diluent, Roche Diagnostics; and Modular Analytics System, Hitachi Ltd.) in blood were measured via electrochemiluminescence immunoassay,^24^, ^25^ and free testosterone in blood was measured by radioimmunoassay (RIA kit; SML Immunotech; γ counter; Hitachi Ltd., PerkinElmer Japan).^24^ SHBG in blood was measured at SRL, Inc. by enzyme‐linked immunosorbent assay^26^ using the DRG SHBG ELISA kit (DRG International) according to the manufacturer's instructions.

The ranges of detection for the biomarkers were 0.11‐249.9 mIU/mL for LH, 0.06‐149.99 mIU/mL for FSH, 4.00‐99,999.99 nmol/L for SHBG, 0.03‐99,900,000 ng/mL for total testosterone, 0.2‐99.9 pg/mL for free testosterone, 5.0‐99,990,000 pg/mL for estradiol, and 0.05‐99,900,000 ng/mL for progesterone.

### Data Analyses

The study protocol called for enrollment of 44 individuals, 16 in Part 1, and 28 in Part 2. This number was considered sufficient for the evaluation of PK, safety, and biomarkers in this study. No formal sample size calculation was performed on the basis of statistical power.

PK analyses were performed using data from individuals who had at least 1 postdose sample taken for drug concentration analysis. Safety analyses were based on data from participants who received at least 1 dose of the study medication, and biomarker analyses were performed using data from individuals who had at least 1 sample taken for biomarker assessment.

PK parameters were estimated by noncompartmental analysis using Phoenix WinNonlin version 7.0 (Certara, LP). Descriptive statistics were evaluated using SAS Drug Development Version 4.5 and SAS version 9.4 (SAS Institute Japan Ltd.). The principal PK parameters investigated in this study after single‐dose administration were area under the concentration–time curve (AUC) from the time of dosing to 24 hours (AUC_24_), AUC from the time of dosing extrapolated to infinity, AUC from the time of dosing to last measurable concentration, apparent total clearance after oral dosing (CL/F), C_max_, metabolite‐to‐parent ratio of AUC_24_, terminal elimination half‐life (t_1/2_), and time to maximum concentration (t_max_). After multiple‐dose administration, the principal parameters were AUC from the time of dosing to start of next dosing interval (AUC_tau_), CL/F, C_max_, metabolite‐to‐parent ratio of AUC_tau_, peak–trough ratio, accumulation ratio), t_1/2_, and t_max_]. The accumulation ratio is the ratio of the AUC_24_ after the first dose and AUC_tau_ at steady state.

For the concentration–QTcF safety assessments, the relationships between fezolinetant or ES259564 and dQTcF were assessed using a linear mixed‐effects model, which has been described previously in a white paper.^27^ The fixed effects on intercept in the model included treatment, population, day of ECG assessment, nominal elapsed time after the last dosing, and baseline QTcF. A random effect was kept on intercept only in the final model. The placebo‐corrected dQTcF (ddQTcF) was then predicted at the geometric mean of C_max_ following 180‐mg dosing on Day 10 using the following equation:

$$\mathrm{ddQTcF}=\theta_{1}\mathrm{TRT}+\theta_{5}\mathrm{fezolinetant}+\theta_{6}\mathrm{ES}259564$$

where: θ_1_ = treatment effect (ms)

$$\begin{array}{ccc} \mathrm{TRT} & = & \mathrm{binary}\;\mathrm{indicator}\;\mathrm{for}\;\mathrm{treatment}\;\\ & & \times \;\left(\mathrm{placebo}=0,\;\mathrm{active}=1\right) \end{array}$$

$$\theta_{5}=\mathrm{slope}\;\mathrm{for}\;\mathrm{fezolinetant}\;\left(\mathrm{ms}/\left(\mu g/\mathrm{mL}\right)\right)$$

$$\theta_{6}=\mathrm{slope}\;\mathrm{for}\;\mathrm{ES}259564\;\left(\mathrm{ms}/\left(\mu g/\mathrm{mL}\right)\right)$$

The International Committee on Harmonization E14 guideline was employed to ascertain whether fezolinetant increased the upper one‐sided 95% confidence interval (CI) for ddQTcF by 10 ms or greater, which would indicate a clinically significant effect.^28^, ^29^ The estimated population mean and the one‐sided 95% CI were calculated by ESTIMATE statement in SAS MIXED procedure.

The LH and FSH biomarker results were calculated and plotted in terms of mean percent change from baseline. For this analysis, baseline was defined as the average of 3 observations on Day ‐1. The principal biomarker parameters for LH and FSH investigated in this study after single‐dose administration were change from baseline in AUC from the time of dosing to 12 hours (AUC_12_b_), AUC_24_b_, AUC from the time of dosing to 48 hours (AUC_48_b_), minimum concentration (C_min_b_), and time of minimum concentration (t_min_b_). After multiple‐dose administration, the principal parameters were change from baseline in AUC_tau_b_, C_min_b_, and t_min_b_. To distinguish these parameters from the similar PK parameters, a subscripted “b” for “biomarker” has been added to these terms. The results for SHBG, total testosterone, free testosterone, estradiol, and progesterone were also calculated and plotted.

## Results

### Study Participants

In Part 1, 16 male participants were randomized, 4 to the placebo group and 6 each to the fezolinetant 15‐ and 60‐mg groups. In Part 2, 28 male and female participants were randomized; 9 male participants to the fezolinetant group and 3 to the placebo group in Cohort 2‐1, 6 premenopausal female participants to the fezolinetant group and two to the placebo group in Cohort 2‐2a, and 6 postmenopausal female participants to the fezolinetant group and two to the placebo group in Cohort 2‐2b. All participants received at least 1 dose of the study drug.

Demographics and baseline characteristics are shown in Table S1. All participants were Japanese. Mean age was similar for the male participants and premenopausal female participants and varied between 22 and 32 years. As expected, postmenopausal female participants were older than premenopausal female participants, and their mean age was approximately 56.4 years. The male participants were slightly heavier than the female participants, although BMI was similar between the sexes. Most of the participants had never smoked.

All randomized participants in Parts 1 and 2 were included in the PK, safety, and biomarker analyses. Two individuals in Part 2 (1 male and 1 postmenopausal female receiving active treatment) discontinued the study before completion owing to TEAEs (unrelated to the study drug); all others completed the study.

### Single‐Dose PK

**Figure 1:**
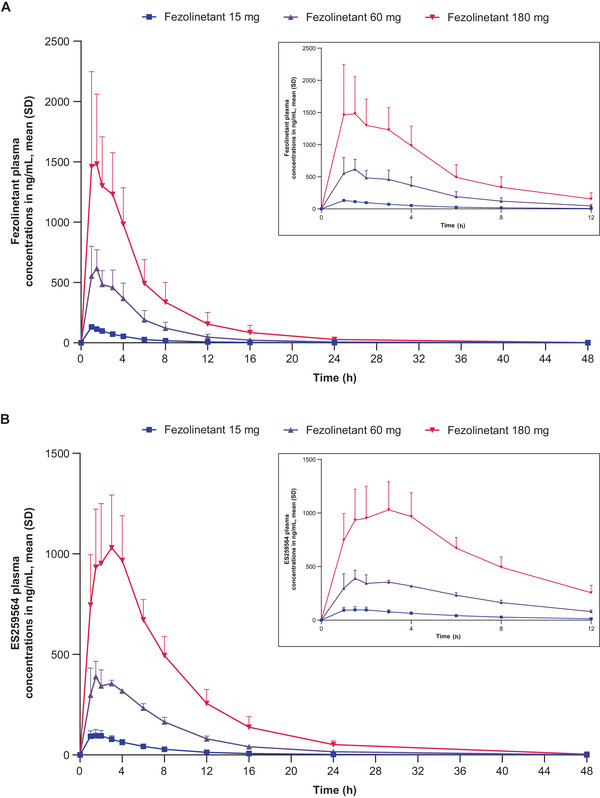
PK results in male participants. Plasma concentration–time profiles of (A) fezolinetant and (B) ES259564 after a single administration of fezolinetant 15 mg (Cohort 1‐1), 60 mg (Cohort 1‐2), or 180 mg (Cohort 2‐1) (Day 1). Insets show time after dosing from 0 to 12. Numbers of participants: fezolinetant 15 mg (Cohort 1‐1): 6, fezolinetant 60 mg (Cohort 1‐2): 6, fezolinetant 180 mg (Cohort 2‐1): 9. Values below the lower limit of quantification (1 ng/mL) were set to zero in the calculation of summary statistics. PK, pharmacokinetic; SD, standard deviation.

**Figure 2:**
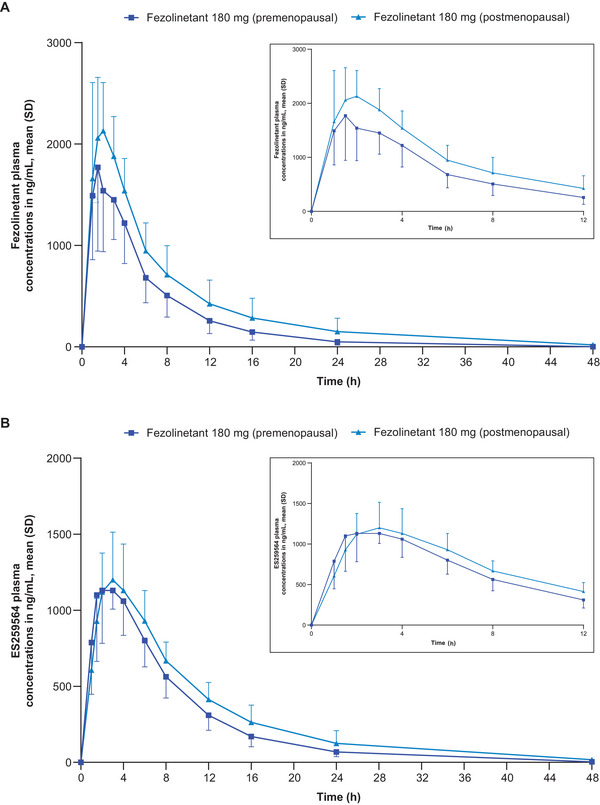
PK results in premenopausal and postmenopausal female participants (cohorts 2‐2a and 2‐2b). Plasma concentration–time profiles of (A) fezolinetant and (B) ES259564 after a single administration of fezolinetant 180 mg (Day 1). Insets show time after dosing from 0 to 12 hours. Numbers of participants: fezolinetant 180 mg (premenopausal; cohort 2‐2a): 6, fezolinetant 180 mg (postmenopausal; cohort 2‐2b): 6. Values below the lower limit of quantification (1 ng/mL) were set to 0 in the calculation of summary statistics. PK, pharmacokinetic; SD, standard deviation.

Plasma concentration–time profiles of fezolinetant and its metabolite ES259564 after single dosing in male and female participants are shown in Figures 1 and 2, respectively, and the related PK parameters are listed in Tables 1 and 2, respectively.

**Table 1:** Summary of Fezolinetant and ES259564 PK Parameters Following Single‐Dose (Treatment Day 1) Fezolinetant 15‐mg (Cohort 1‐1), 60‐mg (Cohort 1‐2), or 180‐mg (Cohort 2‐1) Treatment in Male Participants

	Fezolinetant			ES259564		
PK parameter	15 mg (Cohort 1‐1) (n = 6)	60 mg (Cohort 1‐2) (n = 6)	180 mg (Cohort 2‐1) (n = 9)	Fezolinetant 15 mg (Cohort 1‐1) (n=6)	Fezolinetant 60 mg (Cohort 1‐2) (n = 6)	Fezolinetant 180 mg (Cohort 2‐1) (n = 9)
C_max_ (ng/mL)	135 (19.6)	679 (168)	1790 (485)	103 (28.5)	417 (57.0)	1130 (251)
t_max_ (h)	1.00 (1.00‐1.50)	1.50 (1.00‐3.00)	1.50 (1.00‐3.00)	1.50 (1.00‐2.00)	1.50 (1.00‐3.00)	3.00 (1.50‐4.00)
AUC_24_ (ng•h/mL)	526 (185)	3110 (1060)	8560 (3240)	625 (112)	3040 (270)	8930 (1190)
AUC_inf_ (ng•h/mL)	535 (201)	3140 (1090)	8780 (3400)	644 (112)	3150 (276)	9390 (1280)
AUC_last_ (ng•h/mL)	523 (187)	3110 (1060)	8750 (3400)	625 (112)	3090 (247)	9340 (1260)
t_1/2_ (h)	3.29 (1.51)	3.81 (0.505)	4.93 (1.31)	4.58 (0.976)	5.04 (1.14)	5.85 (1.18)
CL/F (L/h)	30.4 (7.63)	21.5 (8.64)	23.9 (9.87)	—	—	—
MPR	—	—	—	1.24 (0.387)	1.04 (0.381)	1.15 (0.476)

Values are mean (SD) except for t_max_, which is shown as median (range).

AUC_24_, area under the concentration–time curve from the time of dosing to 24 hours; AUC_inf_, area under the concentration–time curve from the time of dosing extrapolated to infinity; AUC_last_, area under the concentration–time curve from the time of dosing to the last measurable concentration; C_max_, maximum concentration; CL/F, apparent total clearance after oral dosing; MPR, metabolite‐to‐parent ratio (molecular weight–corrected AUC_24_ for the metabolite versus AUC_24_ for the parent); PK, pharmacokinetic; SD, standard deviation; t_1/2_, terminal elimination half‐life; t_max_, time to maximum concentration.

**Table 2:** Summary of Fezolinetant and ES259564 PK Parameters Following Single‐Dose (Treatment Day 1) Fezolinetant 180‐mg Treatment in Premenopausal (Cohort 2‐2a) or Postmenopausal (Cohort 2‐2b) Female Participants

	Fezolinetant 180 mg		ES259564	
PK parameter	Premenopausal (Cohort 2‐2a) (n = 6)	Postmenopausal (Cohort 2‐2b) (n = 6)	Premenopausal (Cohort 2‐2a) (n = 6)	Postmenopausal (Cohort 2‐2b) (n = 6)
C_max_ (ng/mL)	1970 (610)	2320 (541)	1320 (257)	1220 (313)
t_max_ (hour)	1.50 (1.00‐4.00)	2.00 (1.00‐3.00)	2.30 (1.50‐4.00)	2.50 (2.00‐3.00)
AUC_24_ (ng•h/mL)	11,200 (3930)	15,900 (5800)	10,300 (1420)	12,100 (2000)
AUC_inf_ (ng•h/mL)	11,600 (4190)	17,600 (7430)	10,900 (1690)	13,600 (2730)
AUC_last_ (ng•h/mL)	11,500 (4200)	17,400 (7180)	10,800 (1680)	13,400 (2600)
t_1/2_ (hour)	4.83 (0.799)	7.24 (1.71)	5.37 (0.860)	7.90 (1.21)
CL/F (L/h)	17.6 (7.28)	11.9 (4.88)	—	—
MPR	—	—	0.967 (0.294)	0.793 (0.250)

Values are mean (SD) except for t_max_, which is shown as median (range).

AUC_24_, area under the concentration–time curve from the time of dosing to 24 hours; AUC_inf_, area under the concentration–time curve from the time of dosing extrapolated to infinity; AUC_last_, area under the concentration–time curve from the time of dosing to last measurable concentration; C_max_, maximum concentration; CL/F, apparent total clearance after oral dosing; MPR, metabolite‐to‐parent ratio (molecular weight–corrected AUC_24_ for the metabolite versus AUC_24_ for the parent); PK, pharmacokinetic; SD, standard deviation; t_1/2_, terminal elimination half‐life; t_max_, time to maximum concentration.

Following single doses of 15, 60, and 180 mg in male and female participants, fezolinetant was rapidly absorbed. On average, C_max_ occurred within 1‐2 hours after dosing. Fezolinetant concentrations declined thereafter in a nearly monophasic manner, with similar t_1/2_ of about 3‐5 hours after doses of 15, 60, and 180 mg in male participants and about 5 and 7 hours after a dose of 180 mg in premenopausal and postmenopausal female participants, respectively. After single doses of fezolinetant 180 mg, mean CL/F was twice as high in male participants compared with postmenopausal female participants.

Dose‐proportional increases in fezolinetant exposure (based on AUC and C_max_) were observed over the range of 15‐180 mg in male participants (Figure S2). Higher exposures were noted in premenopausal and postmenopausal female participants than in male participants treated with single‐dose fezolinetant 180 mg, and exposure was also greater in postmenopausal than premenopausal female participants. Based on mean AUC from the time of dosing extrapolated to infinity results for fezolinetant, mean exposures were respectively 2.0‐fold and 1.3‐fold higher in postmenopausal and premenopausal female participants than in male participants. The PK profile of metabolite ES259564 was similar to that of the parent drug in terms of AUC and C_max_.

### Multiple‐Dose PK

**Figure 3:**
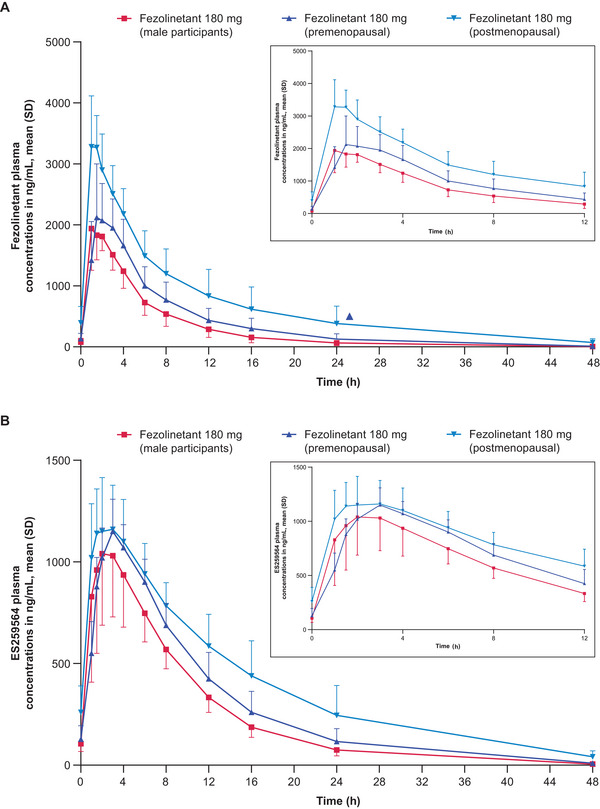
PK results in male participants (Cohort 2‐1) and premenopausal and postmenopausal female participants (Cohorts 2‐2a and 2‐2b). Plasma concentration–time profiles of (A) fezolinetant and (B) ES259564 after multiple administrations of fezolinetant 180 mg (Day 10). Insets show time after dosing from 0 to 12 hours. Numbers of participants: fezolinetant 180 mg (male participants; Cohort 2‐1): 8, fezolinetant 180 mg (premenopausal; cohort 2‐2a): 6, fezolinetant 180 mg (postmenopausal; cohort 2‐2b): 5. Values below the lower limit of quantification (1 ng/mL) were set to zero in the calculation of summary statistics. PK, pharmacokinetic; SD, standard deviation.

Plasma concentration–time profiles of fezolinetant and ES259564 after multiple dosing of 180 mg in male participants and in premenopausal and postmenopausal female participants are shown in Figure 3. Related PK parameters for male and female participants are shown in Table 3.

**Table 3:** Summary of Fezolinetant and ES259564 PK Parameters Following Multiple‐Dose (Treatment Day 10) Fezolinetant 180‐mg Treatment in Male Participants (Cohort 2‐1), Premenopausal Female Participants (Cohort 2‐2a), or Postmenopausal Female Participants (Cohort 2‐2b)

	Fezolinetant 180 mg			ES259564		
PK Parameter	Male participants (Cohort 2‐1) (n = 8)	Premenopausal (Cohort 2‐2a) (n = 6)	Postmenopausal (Cohort 2‐2b) (n = 5)	Male Participants (Cohort 2‐1) (n = 8)	Premenopausal (Cohort 2‐2a) (n = 6)	Postmenopausal (Cohort 2‐2b) (n = 5)
C_max_ (ng/mL)	2170 (436)	2370 (699)	3490 (710)	1110 (350)	1170 (126)	1190 (239)
t_max_ (hour)	1.25 (1.00‐3.00)	1.75 (1.50‐3.00)	1.50 (1.00‐1.50)	2.50 (1.50‐4.00)	3.00(1.50‐3.00)	2.00 (1.00‐3.00)
AUC_tau_ (ng•h/mL)	12,300 (3050)	16,200 (5690)	27,200 (9290)	10,200 (1670)	11,800 (1860)	15,200 (3010)
t_1/2_ (hour)	6.66 (1.13)	6.27 (0.626)	8.98 (2.39)	5.89 (1.26)	6.35 (1.12)	9.11 (2.22)
CL/F (L/h)	15.6 (4.53)	12.4 (4.71)	7.31 (2.56)	—	—	—
PTR	46.6 (57.8)	32.5 (39.7)	16.4 (16.0)	13.1 (10.0)	14.4 (14.7)	5.86 (3.71)
R_ac_(AUC)	1.47 (0.318)	1.46 (0.150)	1.57 (0.151)	1.16 (0.118)	1.14 (0.0573)	1.22 (0.125)
MPR	—	—	—	0.853 (0.336)	0.751 (0.197)	0.572 (0.164)

Values are mean (SD) except for t_max_, which is shown as median (range).

AUC_tau_, area under the concentration–time curve from the time of dosing to start of next dosing interval; C_max_, maximum concentration; CL/F, apparent total clearance after oral dosing; MPR, metabolite‐to‐parent ratio (molecular weight–corrected AUC_tau_ for the metabolite versus AUC_tau_ for the parent); PK, pharmacokinetic; PTR, peak–trough ratio; R_ac_(AUC), accumulation ratio calculated using AUC; SD, standard deviation; t_1/2_, terminal elimination half‐life; t_max_, time to maximum concentration.

The t_max_ for fezolinetant was achieved 1.25‐1.75 hours after dosing in the 3 populations. Mean t_1/2_ values ranged from about 6 to 9 hours among the groups. In terms of clearance, mean CL/F was about twice as high in male participants as in postmenopausal female participants. Exposure was greater in female than male participants (based on AUC_tau_ and C_max_) and in postmenopausal than premenopausal female participants. Mean exposures were respectively 2.2‐ and 1.3‐fold higher in postmenopausal and premenopausal female participants than in male participants.

Mean trough plasma concentrations showed that steady state was reached within 3 days in all groups after once‐daily dosing of fezolinetant 180 mg. Slight accumulations of fezolinetant and the ES259564 metabolite were observed after multiple doses over 10 days in male and female participants.

The PK profile of the ES259564 metabolite was similar to that of the parent molecule in male participants, premenopausal female participants, and postmenopausal female participants.

### Safety

TEAEs were observed in 6 participants during the study: transient creatine phosphokinase elevation (single‐dose fezolinetant 60 mg n = 1), acute sinusitis (single‐dose fezolinetant 180 mg n = 1 in a postmenopausal female participant), upper respiratory tract infection (multiple‐dose placebo n = 1 in a male participant and multiple‐dose fezolinetant 180 mg n = 1 in a male participant), and uterine bleeding (multiple‐dose fezolinetant 180 mg n = 2). No TEAEs were serious or severe, and no deaths occurred. The only TEAE deemed by the investigator to be fezolinetant related was uterine bleeding in two participants treated with multiple‐dose fezolinetant 180 mg: mild metrorrhagia in a premenopausal female participant and mild postmenopausal hemorrhage in a postmenopausal female participant. Two participants stopped treatment because of TEAEs: upper respiratory tract infection in a male participant treated with multiple‐dose fezolinetant 180 mg and moderate acute sinusitis in a postmenopausal female participant who was originally randomized to the multiple‐dose group but actually only received a single dose of fezolinetant 180 mg. There were no clinically relevant changes from baseline in vital signs or laboratory values. The ECG results showed that no participant experienced a QTcF interval change of greater than 60 ms or a QTcF interval value of greater than 450 ms. No clinically significant changes in menstrual cycles were noted for the premenopausal female participants, although a slight menstrual cycle prolongation was observed in 1 individual after fezolinetant administration (42 days).

### Concentration–QTcF Analysis

As shown in Figure S3, the concentration–QTcF analysis showed that the slope achieved was negative for fezolinetant, while it was positive and statistically significant for ES259564. The slopes of the relationship were −1.6703 ms/(µg/mL) for fezolinetant and 5.6913 ms/(µg/mL) for ES259564 (Table S2). The intercept was not significantly different from zero, and all assumed fixed effects on intercept (i.e., day of ECG assessment, population, and treatment) were not significant. In addition, neither nominal time effect for dQTcF measurement was significant. The estimated population mean and its upper one‐sided 95% CI for ddQTcF at the steady‐state C_max_ following fezolinetant 180‐mg dosing in postmenopausal female participants (Cohort 2‐2b), corresponding to a geometric mean C_max_ of 3430 ng/mL for fezolinetant and 1170 ng/mL for ES259564 at steady state, were 2.5554 and 6.5174 ms, respectively. The upper one‐sided 95% CI did not reach the threshold of 10 ms necessary to demonstrate a clinically significant effect.

### Biomarkers

**Figure 4:**
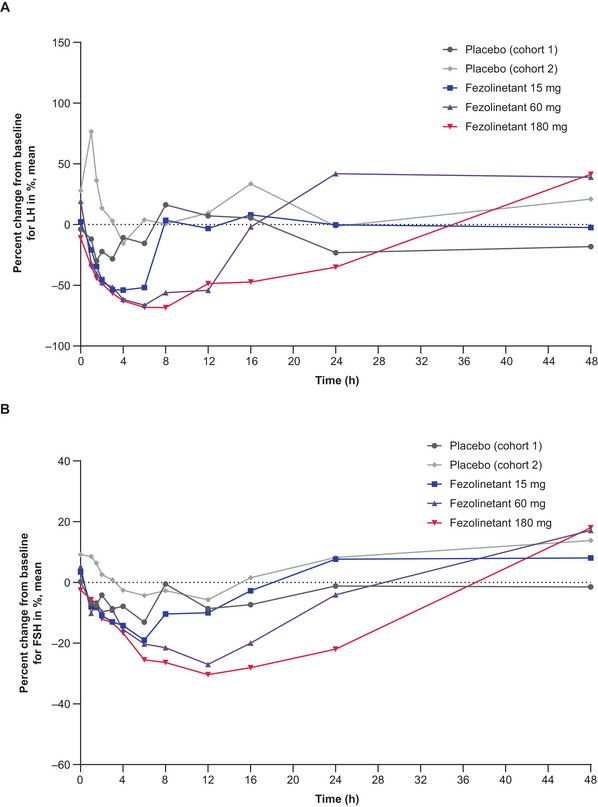
Biomarker results in male participants. Percent change in (A) LH and (B) FSH levels after a single administration of placebo (Cohorts 1 and 2) or fezolinetant 15 mg (Cohort 1‐1), 60 mg (Cohort 1‐2), or 180 mg (Cohort 2‐1) (Day 1). Numbers of participants: placebo (Cohort 1): 4, fezolinetant 15 mg (Cohort 1‐1): 6, fezolinetant 60 mg (Cohort 1‐2): 6, placebo (Cohort 2): 3, fezolinetant 180 mg (Cohort 2‐1): 9. Baseline (time zero) is the mean pharmacodynamic data at 3 time points with at least 2‐h intervals on Day −1. FSH, follicle‐stimulating hormone; LH, luteinizing hormone.

**Figure 5:**
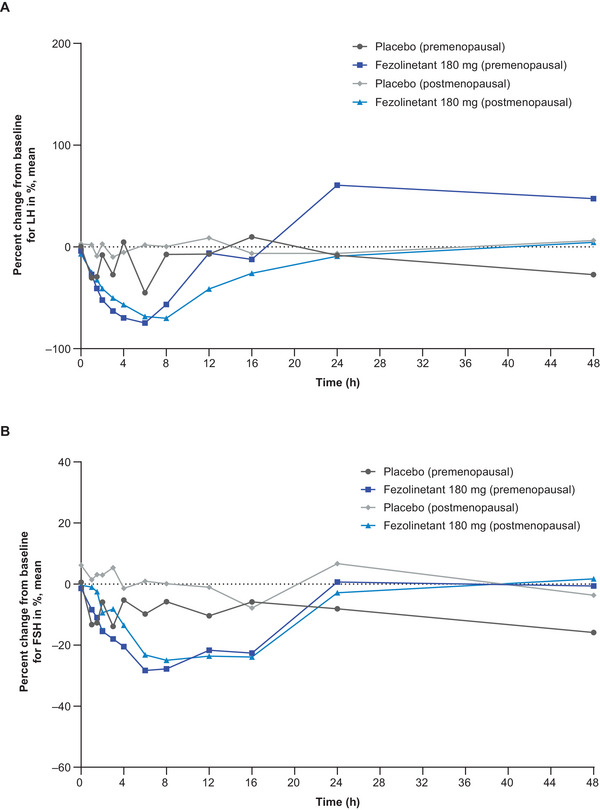
Biomarker results in premenopausal and postmenopausal female participants (cohorts 2‐2a and 2‐2b). Percent change in (A) LH and (B) FSH levels after a single administration of placebo or fezolinetant 180 mg (Day 1). Numbers of participants: placebo (premenopausal; Cohort 2‐2a): two, fezolinetant 180 mg (premenopausal; Cohort 2‐2a): 6, placebo (postmenopausal; Cohort 2‐2b): two, fezolinetant 180 mg (postmenopausal; Cohort 2‐2b): 6. Baseline (time zero) is the mean pharmacodynamic data at 3 time points with at least 2‐h intervals on Day −1. FSH, follicle‐stimulating hormone; LH, luteinizing hormone.

**Figure 6:**
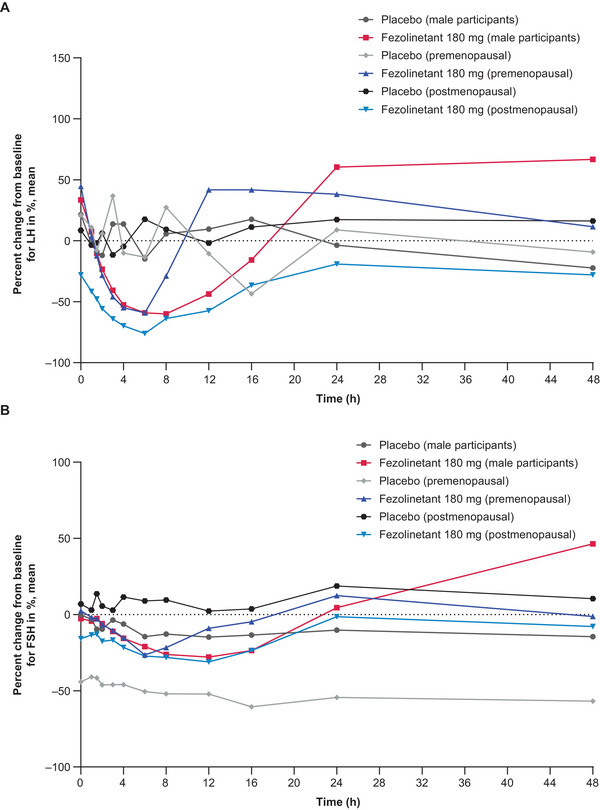
Biomarker results in male participants (Cohort 2‐1) and premenopausal and postmenopausal female participants (Cohorts 2‐2a and 2‐2b). Percent change in (A) LH and (B) FSH levels after multiple administrations of placebo or fezolinetant 180 mg (day 10). Numbers of participants – placebo (male participants; Cohort 2‐1): 3, fezolinetant 180 mg (male participants; Cohort 2‐1): 8, placebo (premenopausal; Cohort 2‐2a): 2, fezolinetant 180 mg (premenopausal; Cohort 2‐2a): 6, placebo (postmenopausal; Cohort 2‐2b): two, fezolinetant 180 mg (postmenopausal; Cohort 2‐2b): 5. Baseline (time zero) is the mean pharmacodynamic data at 3 time points with at least 2‐h intervals on Day −1. FSH, follicle‐stimulating hormone; LH, luteinizing hormone.

The LH and FSH data over time are shown in Figure 4 for male participants after single‐dose administration, Figure 5 for female participants after single‐dose administration, and Figure 6 for male and female participants after multiple‐dose administration. Following the administration of single and multiple doses of fezolinetant, LH concentrations had decreased and returned to baseline or increased from baseline by 48 hours after dosing. FSH concentrations had decreased slightly and returned to baseline or increased from baseline by 24 hours after dosing in both groups of female participants and 48 hours after dosing in male participants treated with 180 mg.

Dose‐dependent decreases in LH concentrations accompanied by small reductions in FSH concentrations were noted in male participants after single‐dose fezolinetant. Maximum reductions in mean LH with 15, 60, and 180 mg were 54%, 66%, and 68% after 4, 6, and 8 hours, respectively. Comparatively, maximum reductions in mean FSH were 19%, 27%, and 30% after 6, 12, and 12 hours, respectively. Similarly, after multiple‐dose fezolinetant 180 mg, male participants had a maximum 60% reduction in mean LH after 8 hours and a maximum 28% reduction in mean FSH after 12 hours. It is also noteworthy that increases in mean LH of 60% and 67% were observed after 24 and 48 hours, respectively.

LH concentrations also decreased after the use of multiple‐dose fezolinetant 180 mg in premenopausal and postmenopausal female participants, while FSH concentrations again declined to a limited extent. For premenopausal female participants in the multiple‐dose fezolinetant 180‐mg group, mean LH was reduced by a maximum of 59%, while mean FSH was reduced by a maximum of 27%, both after 6 hours. In postmenopausal female participants receiving multiple‐dose fezolinetant 180 mg, mean LH was reduced by a maximum of 76% after 6 hours, and mean FSH was reduced by a maximum of 31% after 12 hours. LH and FSH profiles at Day 1 were comparable with those at Day 10 in premenopausal and postmenopausal female participants. Mean LH reduction in postmenopausal female participants was not substantially different from the results observed in premenopausal female participants after multiple doses of fezolinetant 180 mg.

After single‐dose administration in male participants, median t_min_b_ for LH was slightly longer with increasing doses of fezolinetant, although the FSH results were similar for 60  and 180 mg (Table S3). Mean C_min_b_ was typically similar in the placebo and fezolinetant groups for both biomarkers. Compared with placebo, dose‐dependent decreases in AUC_12_b_ and AUC_24_b_ were noted in terms of LH, but the same trend was not always apparent for FSH. After administration of a single dose of fezolinetant to female participants, lower AUC_12_b_, AUC_24_b_, and C_min_b_ results were noted for the postmenopausal versus the premenopausal population for both LH and FSH (Table S4). Furthermore, lower LH and FSH results were obtained for all 3 parameters when the results for fezolinetant were compared with placebo. Following the multiple‐dose period, mean AUC_tau_b_ and C_min_b_ results were lower for both LH and FSH following fezolinetant administration compared with placebo (Table S5). The only exception was the AUC_tau_b_ results for premenopausal female participants, which were higher following fezolinetant treatment. Mean C_min_b_ results for LH were consistently lower for postmenopausal female participants compared with male participants and premenopausal female participants.

The total testosterone, free testosterone, and SHBG data over time are shown in Figure S4 for male participants after single‐dose administration, Figure S5 for female participants after single‐dose administration, and Figure S6 for male and female participants after multiple‐dose administration. Estradiol and progesterone results are also included for the female participants. Total testosterone and free testosterone concentrations were reduced in male participants after all single and multiple doses of fezolinetant and had returned to baseline within 48 hours. Estradiol and progesterone concentrations were highly variable and quantifiable in only a limited number of participants; the only discernible trend appeared to be a slight decrease in estradiol after single‐dose fezolinetant 180 mg in premenopausal female participants. Single‐ and multiple‐dose fezolinetant administration had no clear effect on SHBG concentration in male or female participants.

## Discussion

For any potential therapeutic, it is vital to comprehensively assess the pharmacological properties of the drug in different ethnic populations. As such, this study was the first investigation to describe the PK, safety, tolerability, and biomarker profiles of the NK3R antagonist fezolinetant in a population of healthy Japanese male and female participants.

In this study, fezolinetant was rapidly absorbed and eliminated, with peak concentrations reached after 1‐2 hours and mean t_1/2_ ranging from 6.27 to 8.98 hours after multiple dosing. In addition, clearance was lower after multiple dosing than after single dosing. Exposure was dose proportional (C_max_ and AUC), and there was a slight accumulation of fezolinetant and its metabolite ES259564 following repeated 180‐mg once‐daily doses. The reason for this slight accumulation and decrease in clearance is unclear, as nonclinical study results indicate that fezolinetant does not have any direct or time‐dependent inhibition of CYP1A2, the primary metabolic enzyme for fezolinetant. Female participants had greater fezolinetant exposure than male participants, and postmenopausal female participants appeared to have greater exposure than premenopausal female participants. Although these results may be an artifact of the lower number of participants enrolled in this investigation, the metabolic profile of fezolinetant may also explain these findings. It has been reported that CYP1A2 activity is both higher in males than in females and in younger rather than older individuals.^30^, ^31^ Similar overall results were observed in a previous study conducted in Belgium involving predominantly White (93.8%; Astellas Pharma Inc., study ESN364‐CPK‐101) men and premenopausal women.^20^ In this earlier investigation, fezolinetant had a t_max_ of 3.0‐4.0 hours and a t_1/2_ of 3.9‐7.1 hours after 180‐mg multiple doses in men and women. No significant accumulation or change in PK profile were observed with repeated dosing except for a trend toward increased exposure in women who received 180 mg once daily. While making comparisons across studies are inherently limited, mean C_max_ and total exposure appeared to be slightly greater in Japanese male participants compared with Belgian men across the range of doses studied following single and multiple administrations.^20^ Overall, the results of this study can be used as a starting point to identify the optimal dose for future evaluation in Japanese individuals, which will be assessed in subsequent population PK late‐phase clinical studies.

The current study showed that single and multiple doses of fezolinetant had an acceptable safety profile in Japanese male and female participants when administered at doses of up to 180 mg per day. This finding is consistent with the results of the above study conducted in Belgium.^20^ In our investigation, the only fezolinetant‐related TEAE was mild uterine bleeding in 1 premenopausal and 1 postmenopausal female participant, and no fezolinetant‐related serious TEAEs were noted in the previous study. Both studies showed that no safety and tolerability concerns were apparent following fezolinetant dosing. There were no participants who had QTcF interval value above 450 ms or a change from baseline above 60 ms in this study. Exploratory modeling for the concentration–QTcF relationship also found that a negative slope was apparent for fezolinetant, and a positive slope was noted for the metabolite. The estimated upper one‐sided 95% CI for ddQTcF did not exceed 10 ms at the maximum dose of 180 mg, although the quantity of data and the tested dosing range are limited in this study. The observed slope trends and modeling results were consistent with another modeling study that was conducted using data from a non‐Japanese study population and supratherapeutic doses of up to 900 mg (Astellas Pharma Inc., study ESN364‐CPK‐102). The predictions from this previous modeling study suggested that there was no clinically relevant prolongation of QTcF at 8 times the maximum approved therapeutic dose of 45 mg. This modeling, in conjunction with the similar trends identified in the current study, indicates that fezolinetant does not cause QT prolongation in the Japanese population at therapeutic doses.

The biomarker assessments conducted in this study showed that decreases in LH concentrations and small decreases in FSH concentrations were noted following fezolinetant treatment. The levels had returned to baseline by the end of each of the sampling periods. The proportional reductions in LH were not substantially different for premenopausal and postmenopausal female participants after multiple doses of fezolinetant 180 mg. Consistent with the biomarker results presented here, previous clinical studies that involved predominantly White postmenopausal women, as well as men and premenopausal women, have shown that fezolinetant decreases plasma LH but has a limited effect on FSH concentrations.^9^, ^10^, ^20^ These studies also showed that fezolinetant has no clear effect on estradiol or SHBG levels in postmenopausal women.^9^, ^10^ Furthermore, the first‐in‐human study of fezolinetant found that decreases in estradiol and progesterone were observed in premenopausal women as well as testosterone decreases in men.^20^

This study was conducted to evaluate the PK, safety/tolerability, and biomarker profile of fezolinetant in Japanese individuals. As a common limitation of Phase 1 trials, this Phase 1 dose study enrolled only a small number of individuals in each treatment group, and each participant received a maximum of only 10 days of treatment. However, the results of this investigation provided PK and safety data that were critical for informing and enabling the study design for a Phase 2 study in the Japanese population. Further investigations involving a larger number of individuals who receive treatment for a longer duration are required to fully characterize the efficacy and safety profile of fezolinetant in individuals with VMS with differing ethnic backgrounds.

## Conclusion

This investigation demonstrated that the NK3R antagonist fezolinetant was rapidly absorbed and demonstrated dose‐proportional exposure after single doses of up to 180 mg in a Japanese population of healthy male participants and premenopausal and postmenopausal female participants. Fezolinetant had an acceptable safety profile following once‐daily dosing of up to 180 mg and had no clinically relevant effects on QTcF prolongation. Consistent with previous findings in White individuals, lower LH levels and slightly decreased FSH concentrations were noted following fezolinetant treatment. The results of the current study add to the weight of evidence on the safety, efficacy, PK, and biomarker profile of fezolinetant and indicates that the drug would be similarly safe in Japanese individuals for the treatment of VMS due to menopause.

## Conflicts of Interest

Akira Koibuchi, Megumi Iwai, Kanji Komatsu, Kentaro Kuroishi, Mai Shibata, Masako Saito, Jace Nielsen, and Jiayin Huang are employees, or former employees, of Astellas Pharma Inc. Akira Koibuchi, Megumi Iwai, and Mai Shibata hold stock in Astellas Pharma Inc. Jiayin Huang is currently an employee of Amgen Inc. Shunji Matsuki has no further conflict of interest outside of the submitted work.

## Funding

This study was funded by Astellas Pharma Inc. As current or former employees of Astellas Pharma Inc., Akira Koibuchi, Megumi Iwai, Kanji Komatsu, Kentaro Kuroishi, Mai Shibata, Masako Saito, Jace Nielsen, and Jiayin Huang provided substantial contributions to the conception or design of the studies or the acquisition, analysis, or interpretation of data for the studies; drafted the manuscript or reviewed it critically for important intellectual content; and provided final approval of the version to be submitted.

## Supporting information



Supporting Information

## Data Availability

Details for how researchers may request access to anonymized participant level data, trial‐level data, and protocols from Astellas sponsored clinical trials can be found at https://www.clinicaltrials.astellas.com/transparency/.
