# Supplementary material for: Pharmacokinetics, Tolerability, and Biomarker Profile of the Neurokinin 3 Receptor Antagonist Fezolinetant in Healthy Japanese Individuals: A 2‐Part, Randomized, Phase 1 Study
Source: Clin Pharmacol Drug Dev. 2025 Sep 12;14(12):934–50. doi: 10.1002/cpdd.1593 (PMC12672408; doi:10.1002/cpdd.1593)
Supplement: Supplementary file 1 — Supporting Information [file CPDD-14-934-s001.pdf]

Pharmacokinetics, Tolerability, and Biomarker Profile of the Neurokinin 3 Receptor Antagonist Fezolinetant in Healthy Japanese Individuals: A Two-Part, Randomized, Phase 1 Study

Supplementary Materials

Table S1. Demographics and Baseline Characteristics

Table S2. Estimations from the Linear Mixed Effects Model Investigating the Relationship Between Fezolinetant and ES259564 Concentrations and dQTcF

Table S3. Change from Baseline in Biomarker Parameters Following Single-Dose (Treatment Day 1) Administration of Placebo (Cohorts 1 and 2) or Fezolinetant 15 mg (Cohort 1-1), 60 mg (Cohort 1-2), or 180 mg (Cohort 2-1) to Male Participants

Table S4. Change from Baseline in Biomarker Parameters Following Single-Dose (Treatment Day 1) Administration of Placebo or Fezolinetant 180 mg to Premenopausal Female Participants (Cohort 2-2a) or Postmenopausal Female Participants (Cohort 2-2b)

Table S5. Change from Baseline in Biomarker Parameters Following Multiple-Dose (Treatment Day 10) Administration of Placebo or Fezolinetant 180 mg to Male Participants (Cohort 2-1), Premenopausal Female Participants (Cohort 2-2a), or Postmenopausal Female Participants (Cohort 2-2b)

Figure S1. Structures of (A) fezolinetant and (B) ES259564

Figure S2. Dose proportionality of fezolinetant after a single administration of fezolinetant 15 mg (cohort 1-1), 60 mg (cohort 1-2), or 180 mg (cohort 2-1) by (A) AUC_{inf}, (B) AUC_{last}, and (C) C_{max}.

Figure S3. Scatter plots of (A) fezolinetant and (B) ES259564 concentrations against dQTcF intervals

Figure S4. Biomarker results in male participants. Percent change in (A) TT, (B) FT, and (C) SHBG levels after a single administration of placebo (cohorts 1 and 2) or fezolinetant 15 mg (cohort 1-1), 60 mg (cohort 1-2), or 180 mg (cohort 2-1) (day 1)

Figure S5. Biomarker results in premenopausal and postmenopausal female participants (cohorts 2-2a and 2-2b). Percent change in (A) TT, (B) FT, (C) SHBG, (D) estradiol, and (E) progesterone levels after a single administration of placebo or fezolinetant 180 mg (Day 1)

Figure S6. Biomarker results in male participants (cohort 2-1) and premenopausal and postmenopausal female participants (cohorts 2-2a and 2-2b). Percent change in (A) TT, (B) FT, (C) SHBG, (D) estradiol, and (E) progesterone levels after multiple administrations of placebo or fezolinetant 180 mg (Day 10)

Table S1. Demographics and Baseline Characteristics

Part	Cohort	Population	Treatment	n	Age (y)	Weight (kg)	BMI (kg/m^2)[a]
1 (single-dose cohort)	Total of 1-1 and 1-2	Male participants	Placebo	4	21.8 (21–22)	59.35 (52.1–66.6)	21.00 (18.7–22.8)
			Fezolinetant	12	22.1 (20–34)	61.87 (53.5–75.6)	20.83 (18.3–25.8)
2 (multiple-dose cohort)	2-1	Male participants	Placebo	3	28.7 (26–31)	68.10 (56.5–78.1)	23.23 (18.8–25.9)
			Fezolinetant	9	32.2 (22–39)	65.19 (52.6–72.5)	22.09 (17.6–25.6)
	2-2a	Premenopausal female participants	Placebo	2	27.5 (26–29)	56.80 (53.6–60.0)	21.75 (20.2–23.3)
			Fezolinetant	6	29.5 (23–36)	54.88 (49.5–64.3)	22.08 (18.5–25.0)
	2-2b	Postmenopausal female participants	Placebo	2	56.5 (52–61)	54.95 (49.2–60.7)	22.90 (19.8–26.0)
			Fezolinetant	6	56.3 (52–62)	52.73 (44.8–67.8)	21.25 (17.6–26.3)

Values are mean (range).

BMI, body mass index.

[a]BMI at screening.

Table S2. Estimations from the Linear Mixed Effects Model Investigating the Relationship Between Fezolinetant and ES259564 Concentrations and dQTcF

	Parameter (unit)	Estimate	Standard error	*P* value
Θ_0	Intercept (msec)	1.1752	1.7177	0.4961
Θ_1	Treatment effect (msec)	1.6257	1.6293	0.3228
Θ_2	Effect of premenopausal female on intercept (msec)	–0.5603	2.1236	0.7932
Θ_3	Effect of postmenopausal female on intercept (msec)	–2.1533	2.0094	0.2892
Θ_4	Additional intercept for day 10 (msec)	0.7522	1.5552	0.6293
Θ_5	Slope for fezolinetant (msec/(µg/mL))	–1.6703	1.1605	0.1516
Θ_6	Slope for ES259564 (msec/(µg/mL))	5.6913	2.5771	0.0283
Θ_7	QTcF baseline effect (msec)	–0.07095	0.04715	0.1401
	Nominal time 0 hours (msec)[a]	—	—	—
Θ_8	Nominal time 1 hours (msec)	–2.8728	1.4762	0.0530
Θ_9	Nominal time 2 hours (msec)	–0.9695	1.4931	0.5169
Θ_{10}	Nominal time 4 hours (msec)	–0.1296	1.4988	0.9312
Θ_{11}	Nominal time 24 hours (msec)	–1.1345	1.4678	0.4404
$\eta_{\Theta 0}$	Inter-individual variability of intercept (msec^2)	11.5304		
ε	Residual variance (msec^2)	27.4525		

dQTcF, change from baseline in QT intervals corrected for heart rate using Fridericia's formula.

[a]Reference of nominal time effect (j = 1).

$$dQTcF_{ij} = \left(\theta_0 + \eta_{\theta_0,i} + \theta_1 TRT_i + \theta_2 PRE_i + \theta_3 POST_i + \theta_4 DAY_i\right) + \theta_5 fezolinetant_{ij} + \theta_6 ES259564_{ij} + \theta_7\left(QTcF_{i,0} - \overline{QTcF_0}\right) + \sum_{j=2}^{5} \theta_{(j+6)} T_j + \varepsilon_{ij}$$

where:

$\theta_{(j+6)}$ = the parameter estimate associated with the jth nominal time

TRT = binary indicator for treatment (placebo = 0, active = 1)

PRE = population (premenopausal female participant = 1, others = 0)

$POST$ = population (postmenopausal female participant = 1, others = 0)

DAY = day of electrocardiogram (ECG) assessment (day 1 = 0, day 10 = 1)

$fezolinetant$ = plasma fezolinetant concentration (ng/mL)

$ES259564$ = plasma ESN259564 concentration (ng/mL)

$QTcF_{i,0}$ = individual baseline QT interval corrected for heart rate using Fridericia's formula (QTcF)

$\overline{QTcF_0}$ = grand mean baseline QTcF interval for all participants

T_j = binary indicator for the jth nominal time

$\eta_{\theta_{0,i}}$ = random effect on intercept

ε_{ij} = residual error

Table S3. Change from Baseline in Biomarker Parameters Following Single-Dose (Treatment Day 1) Administration of Placebo (Cohorts 1 and 2) or Fezolinetant 15 mg (Cohort 1-1), 60 mg (Cohort 1-2), or 180 mg (Cohort 2-1) to Male Participants

Parameter	Placebo (cohort 1) (n = 4)	Fezolinetant 15 mg (cohort 1-1) (n = 6)	Fezolinetant 60 mg (cohort 1-2) (n = 6)	Placebo (cohort 2) (n = 3)	Fezolinetant 180 mg (cohort 2-1) (n = 9)
**LH**					
AUC_{12_b} (mIU·h/mL)	–4.86 (12.6)	–12.1 (16.9)	–21.4 (11.9)	3.54 (4.85)	–21.6 (9.29)
AUC_{24_b} (mIU·h/mL)	–10.7 (28.1)	–12.7 (28.2)	–23.2 (35.5)	9.21 (6.38)	–37.9 (21.6)
AUC_{48_b} (mIU·h/mL)	–40.3 (61.9)	–15.5 (38.7)	1.92 (74.7)	11.6 (20.5)	–36.0 (35.7)
C_{min_b} (mIU/mL)	–2.47 (1.44)	–2.06 (1.16)	–2.30 (0.794)	–0.849 (0.510)	–2.26 (0.675)
t_{min_b} (h)	7.50 (1.50–24.0)	4.00 (3.00–6.00)	7.00 (4.00–12.0)	8.00 (4.00–24.0)	12.0 (6.00–24.0)
**FSH**					
AUC_{12_b} (mIU·h/mL)	–2.83 (2.38)	–3.59 (5.27)	–4.61 (2.88)	–0.869 (2.09)	–8.76 (5.07)
AUC_{24_b} (mIU·h/mL)	–5.00 (3.17)	–4.24 (10.1)	–9.20 (7.46)	–0.676 (5.49)	–19.4 (9.84)
AUC_{48_b} (mIU·h/mL)	–6.07 (3.54)	–0.419 (16.7)	–7.07 (13.7)	5.63 (14.7)	–21.7 (16.7)
C_{min_b} (mIU/mL)	–0.513 (0.188)	–0.483 (0.427)	–0.629 (0.330)	–0.239 (0.180)	–1.13 (0.510)
t_{min_b} (h)	6.00 (3.00–12.0)	6.00 (6.00–12.0)	12.0 (6.00–16.0)	12.0 (8.00–16.0)	12.0 (6.00–24.0)

Values are mean (SD) except for t_{min_b}, which is shown as median (range).

To distinguish these parameters from the similar pharmacokinetic parameters, a subscripted "b" for "biomarker" has been added to these terms.

AUC_{12_b}, area under the concentration-time curve (AUC) from the time of dosing to 12 hours; AUC_{24_b}, AUC from the time of dosing to 24 hours; AUC_{48_b}, AUC from the time of dosing to 48 hours; C_{min_b}, minimum concentration; FSH, follicle-stimulating hormone; LH, luteinizing hormone; SD, standard deviation; t_{min_b}, time of minimum concentration.

Table S4. Change from Baseline in Biomarker Parameters Following Single-Dose (Treatment Day 1) Administration of Placebo or Fezolinetant 180 mg to Premenopausal Female Participants (Cohort 2-2a) or Postmenopausal Female Participants (Cohort 2-2b)

Parameter	Premenopausal Placebo (cohort 2-2a) (n = 2)	Premenopausal Fezolinetant 180 mg (cohort 2-2a) (n = 6)	Postmenopausal Placebo (cohort 2-2b) (n = 2)	Postmenopausal Fezolinetant 180 mg (cohort 2-2b) (n = 6)
**LH**				
AUC_{12_b} (mIU·h/mL)	–3.97 (NC)	–21.0 (14.9)	2.31 (NC)	–138 (38.3)
AUC_{24_b} (mIU·h/mL)	–4.08 (NC)	–20.3 (28.0)	–9.66 (NC)	–194 (85.7)
AUC_{48_b} (mIU·h/mL)	–16.8 (NC)	–3.55 (45.3)	–8.92 (NC)	–196 (79.8)
C_{min_b} (mIU/mL)	–1.17 (NC)	–2.62 (1.39)	–4.41 (NC)	–16.2 (4.75)
t_{min_b} (h)	27.0 (6.00–48.0)	7.00 (4.00–16.0)	12.8 (1.50–24.0)	10.0 (6.00–16.0)
**FSH**				
AUC_{12_b} (mIU·h/mL)	–6.63 (NC)	–18.7 (17.4)	7.46 (NC)	–111 (35.2)
AUC_{24_b} (mIU·h/mL)	–13.4 (NC)	–32.6 (33.3)	–10.6 (NC)	–215 (85.6)
AUC_{48_b} (mIU·h/mL)	–31.9 (NC)	–44.4 (60.5)	15.4 (NC)	–206 (209)
C_{min_b} (mIU/mL)	–0.950 (NC)	–2.41 (2.02)	–6.21 (NC)	–16.0 (3.68)
t_{min_b} (h)	24.8 (1.50–48.0)	8.00 (6.00–16.0)	32.0 (16.0–48.0)	10.0 (6.00–16.0)

Values are mean (SD) except for t_{min_b}, which is shown as median (range).

To distinguish these parameters from the similar pharmacokinetic parameters, a subscripted "b" for "biomarker" has been added to these terms.

AUC_{12_b}, area under the concentration-time curve (AUC) from the time of dosing to 12 hours; AUC_{24_b}, AUC from the time of dosing to 24 hours; AUC_{48_b}, AUC from the time of dosing to 48 hours; C_{min_b}, minimum concentration; FSH, follicle-stimulating hormone; LH, luteinizing hormone; NC, not calculable; SD, standard deviation; t_{min_b}, time of minimum concentration.

Table S5. Change from Baseline in Biomarker Parameters Following Multiple-Dose (Treatment Day 10) Administration of Placebo or Fezolinetant 180 mg to Male Participants (Cohort 2-1), Premenopausal Female Participants (Cohort 2-2a), or Postmenopausal Female Participants (Cohort 2-2b)

Parameter	Male participants Placebo (cohort 2-1) (n = 3)	Male participants Fezolinetant 180 mg (cohort 2-1) (n = 8)	Premenopausal Placebo (cohort 2-2a) (n = 2)	Premenopausal Fezolinetant 180 mg (cohort 2-2a) (n = 6)	Postmenopausal Placebo (cohort 2-2b) (n = 2)	Postmenopausal Fezolinetant 180 mg (cohort 2-2b) (n = 5)
**LH**						
AUC_{tau_b} (mIU·h/mL)	3.02 (13.7)	–13.3 (18.2)	–6.07 (NC)	4.65 (41.7)	50.4 (NC)	–230 (111)
C_{min_b} (mIU/mL)	–0.986 (0.679)	–1.95 (0.606)	–1.28 (NC)	–1.96 (1.15)	–3.24 (NC)	–15.8 (4.02)
t_{min_b} (h)	48.0 (8.00–48.0)	8.00 (4.00–12.0)	20.0 (16.0–24.0)	5.00 (3.00–6.00)	2.00 (1.00–3.00)	12.0 (6.00–12.0)
**FSH**						
AUC_{tau_b} (mIU·h/mL)	–8.64 (10.0)	–12.9 (12.0)	–73.4 (NC)	–67.4 (154)	140 (NC)	–253 (190)
C_{min_b} (mIU/mL)	–0.582 (0.584)	–0.994 (0.602)	–3.42 (NC)	–3.64 (6.18)	–1.67 (NC)	–16.6 (8.42)
t_{min_b} (h)	12.0 (8.00–48.0)	12.0 (8.00–16.0)	16.0 (16.0–16.0)	6.00 (6.00–48.0)	9.50 (3.00–16.0)	12.0 (6.00–16.0)

Values are mean (SD) except for t_{min_b}, which is shown as median (range).

To distinguish these parameters from the similar pharmacokinetic parameters, a subscripted “b” for “biomarker” has been added to these terms.

AUC_{tau_b}, area under the concentration-time curve (AUC) from the time of dosing to start of next dosing interval; C_{min_b}, minimum concentration; FSH, follicle-stimulating hormone; LH, luteinizing hormone; NC, not calculable; SD, standard deviation; t_{min_b}, time of minimum concentration.

Figure S1. Structures of (A) fezolinetant and (B) ES259564

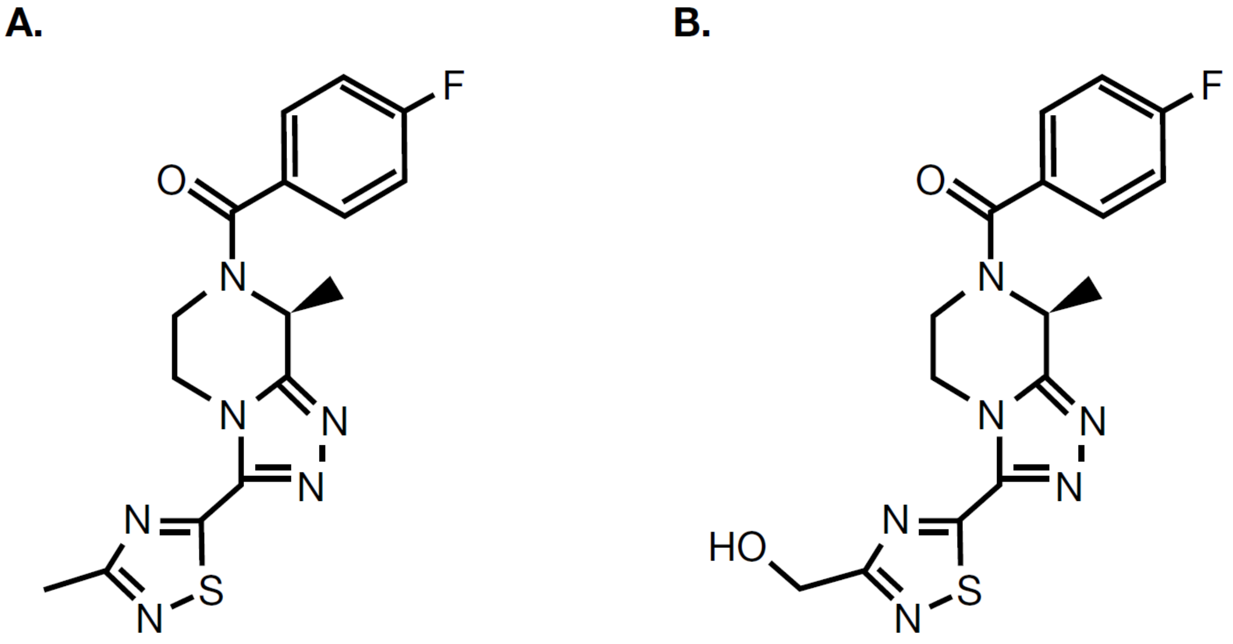

Figure S2. Dose proportionality of fezolinetant after a single administration of fezolinetant 15 mg (cohort 1-1), 60 mg (cohort 1-2), or 180 mg (cohort 2-1) by (A) AUC_{inf}, (B) AUC_{last}, and (C) C_{max}

A.

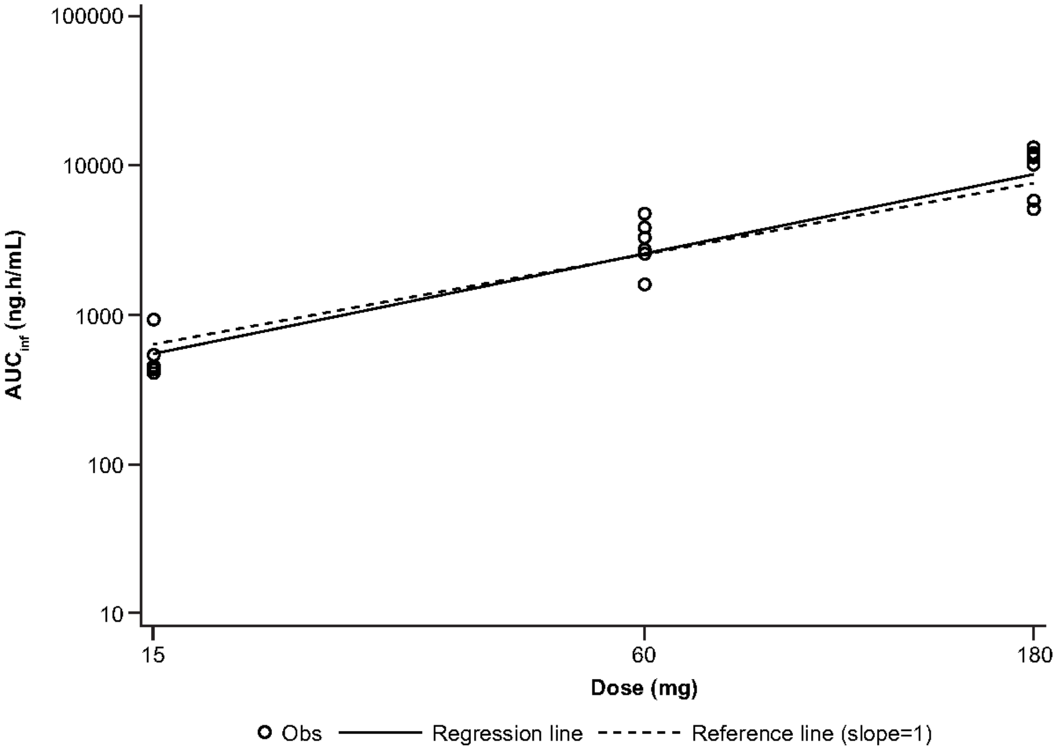

B.

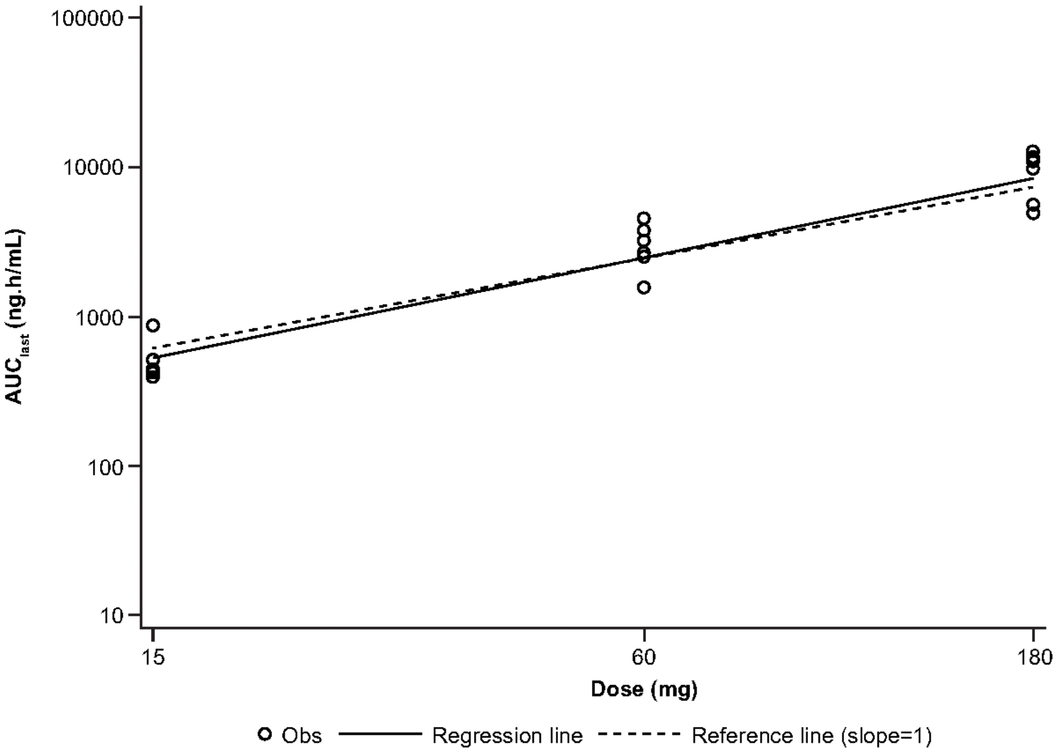

C.

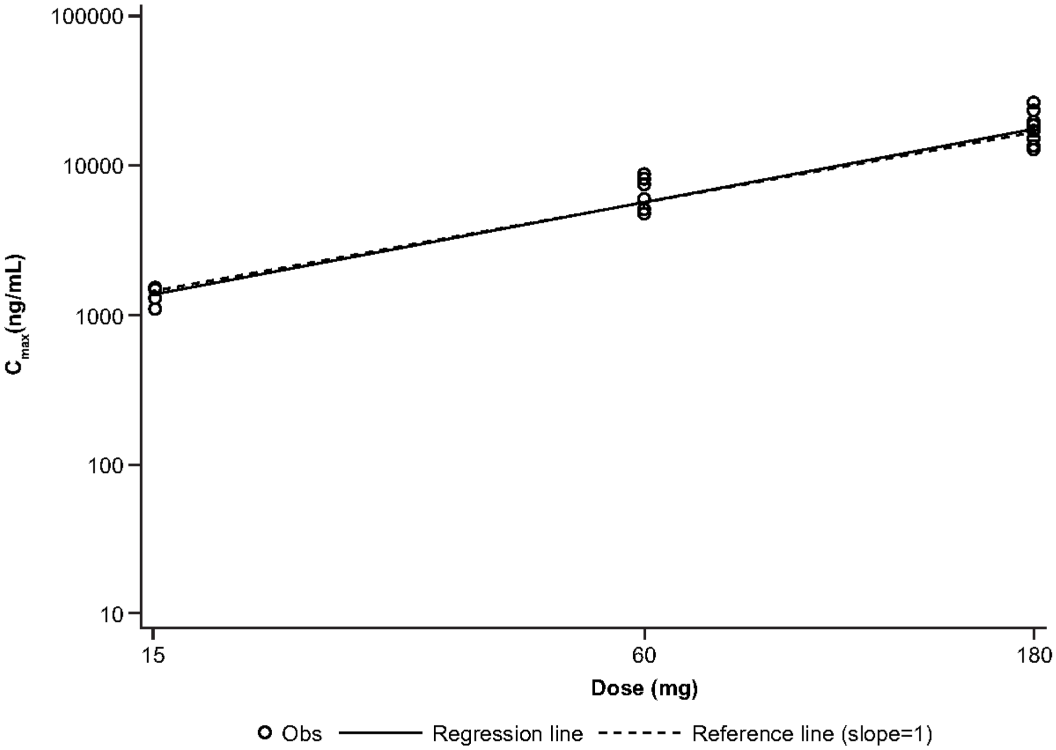

Dose proportionality of fezolinetant was visually assessed using the double-logarithmic plot of PK parameters against dose.

Reference line shows slope=1 in the following regression:

$\ln(\text{PK parameter}) = \alpha + \beta \cdot \ln(\text{dose})$

where α and β represent the intercept and the slope of the regression line, respectively.

Figure S3. Scatter plots of (A) fezolinetant and (B) ES259564 concentrations against dQTcF intervals

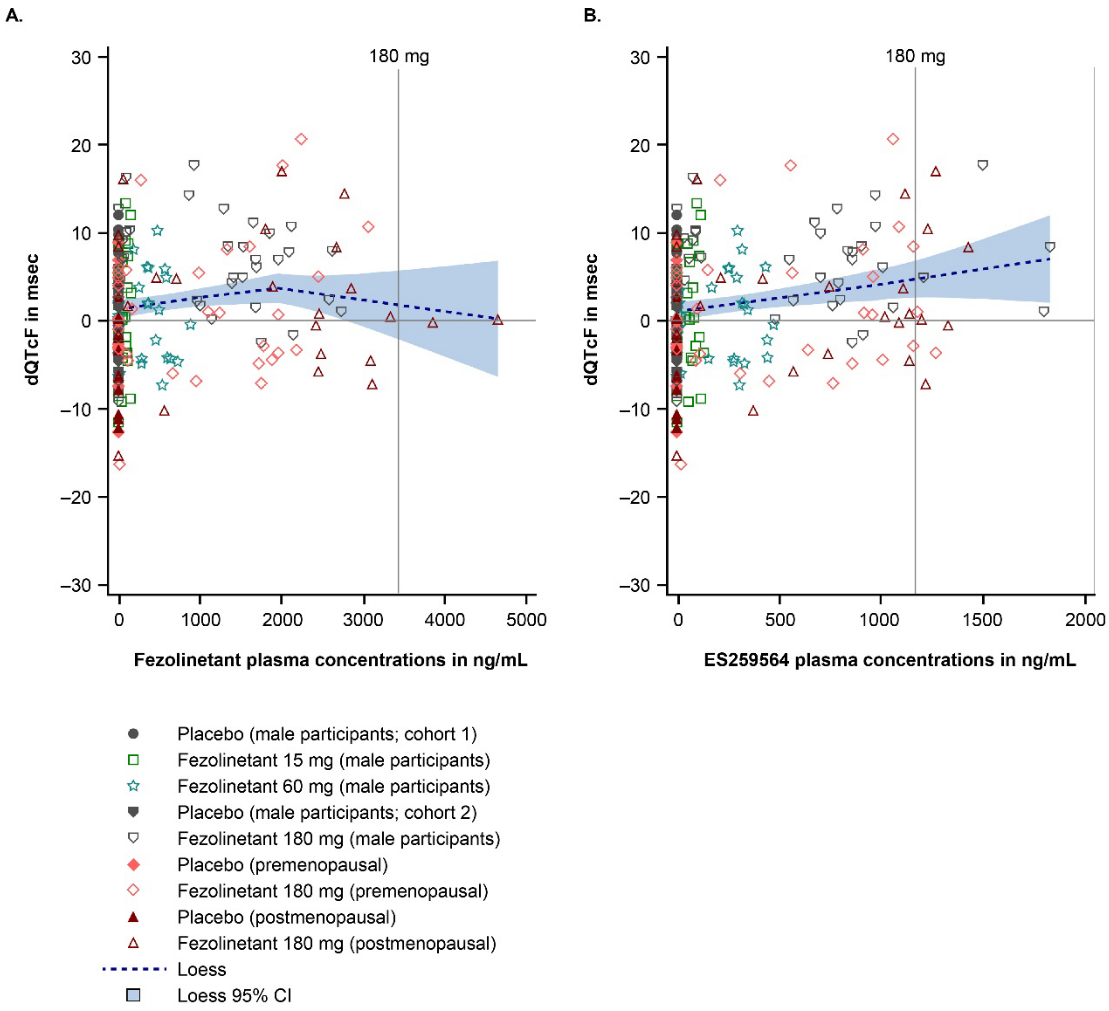

Dotted blue line shows loess smoothing of the observations. The blue band is the 95% CI of the loess line.

Vertical reference line shows the geometric mean for C_{max} in part 2, day 10 (3430 ng/mL and 1170 ng/mL for fezolinetant and ES259564, respectively).

Data for part 1 were acquired after the administration of the single dose and data for part 2 were acquired after 10 days of dosing.

Numbers of participants – placebo (male participants; cohort 1): four, fezolinetant 15 mg (male participants; cohort 1-1): six, fezolinetant 60 mg (male participants; cohort 1-2): six, placebo (male participants; cohort 2): three, fezolinetant 180 mg (male participants; cohort 2-1): nine, placebo (premenopausal; cohort 2-2a): two, fezolinetant 180 mg (premenopausal; cohort 2-2a): six, placebo (postmenopausal; cohort 2-2b): two, fezolinetant 180 mg (postmenopausal; cohort 2-2b): six.

CI, confidence interval; C_{max}, maximum concentration; dQTcF, change from baseline in QT intervals corrected for heart rate using Fridericia's formula.

Figure S4. Biomarker results in male participants. Percent change in (A) TT, (B) FT, and (C) SHBG levels after a single administration of placebo (cohorts 1 and 2) or fezolinetant 15 mg (cohort 1-1), 60 mg (cohort 1-2), or 180 mg (cohort 2-1) (day 1)

A.

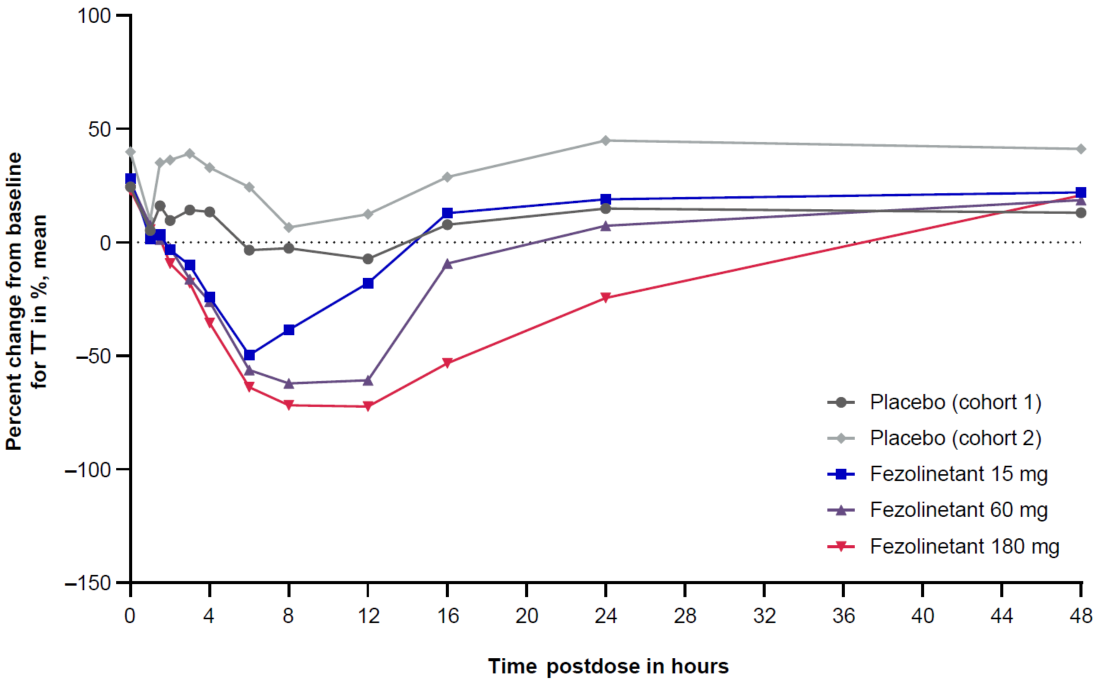

B.

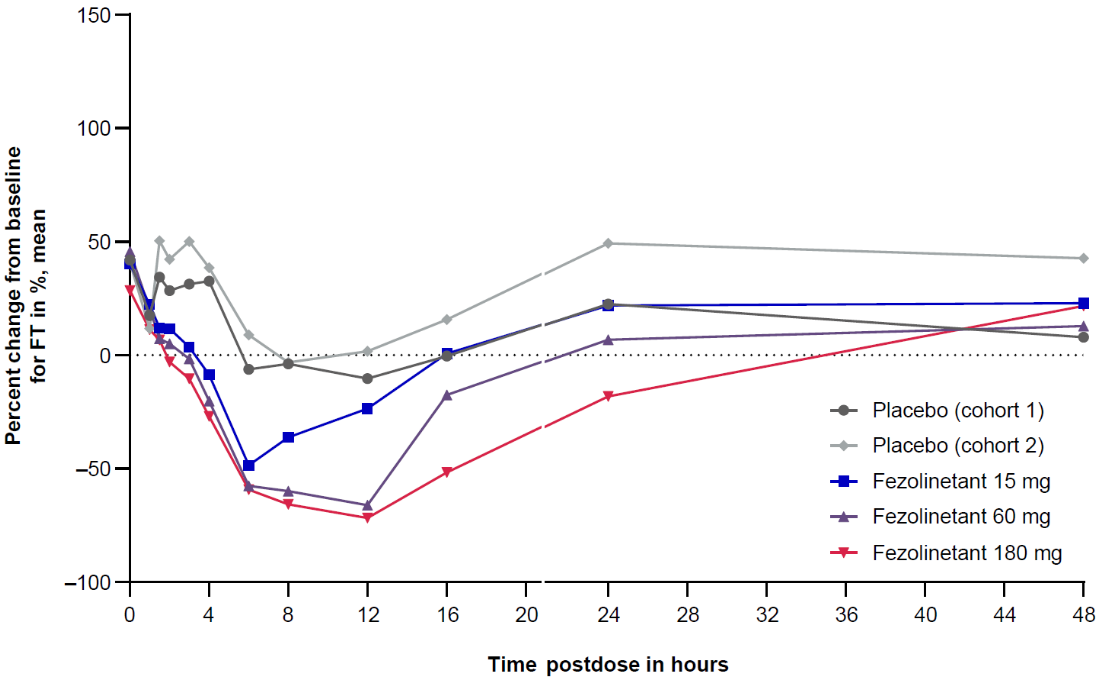

C.

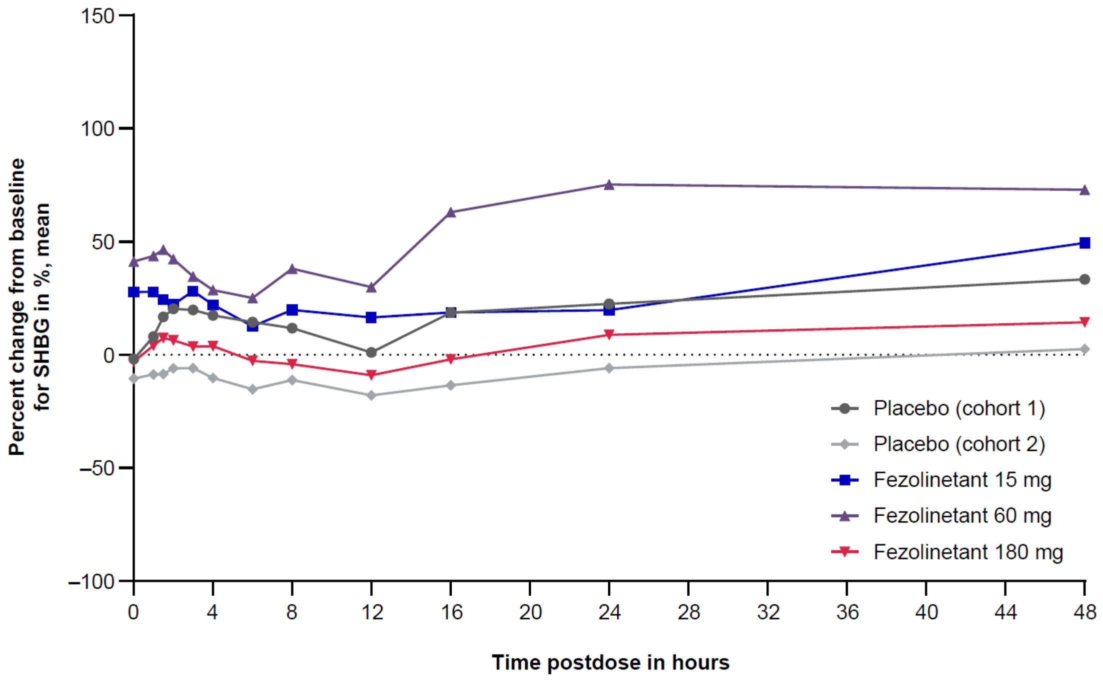

Numbers of participants – placebo (cohort 1): four, fezolinetant 15 mg (cohort 1-1): six, fezolinetant 60 mg (cohort 1-2): six, placebo (cohort 2): three, fezolinetant 180 mg (cohort 2-1): nine.

Baseline (time 0) is the mean pharmacodynamic data at three time points with at least two-hour intervals on Day −1.

FT, free testosterone; SD, standard deviation; SHBG, sex hormone binding globulin; TT, total testosterone.

Figure S5. Biomarker results in premenopausal and postmenopausal female participants (cohorts 2-2a and 2-2b). Percent change in (A) TT, (B) FT, (C) SHBG, (D) estradiol, and (E) progesterone levels after a single administration of placebo or fezolinetant 180 mg (Day 1)

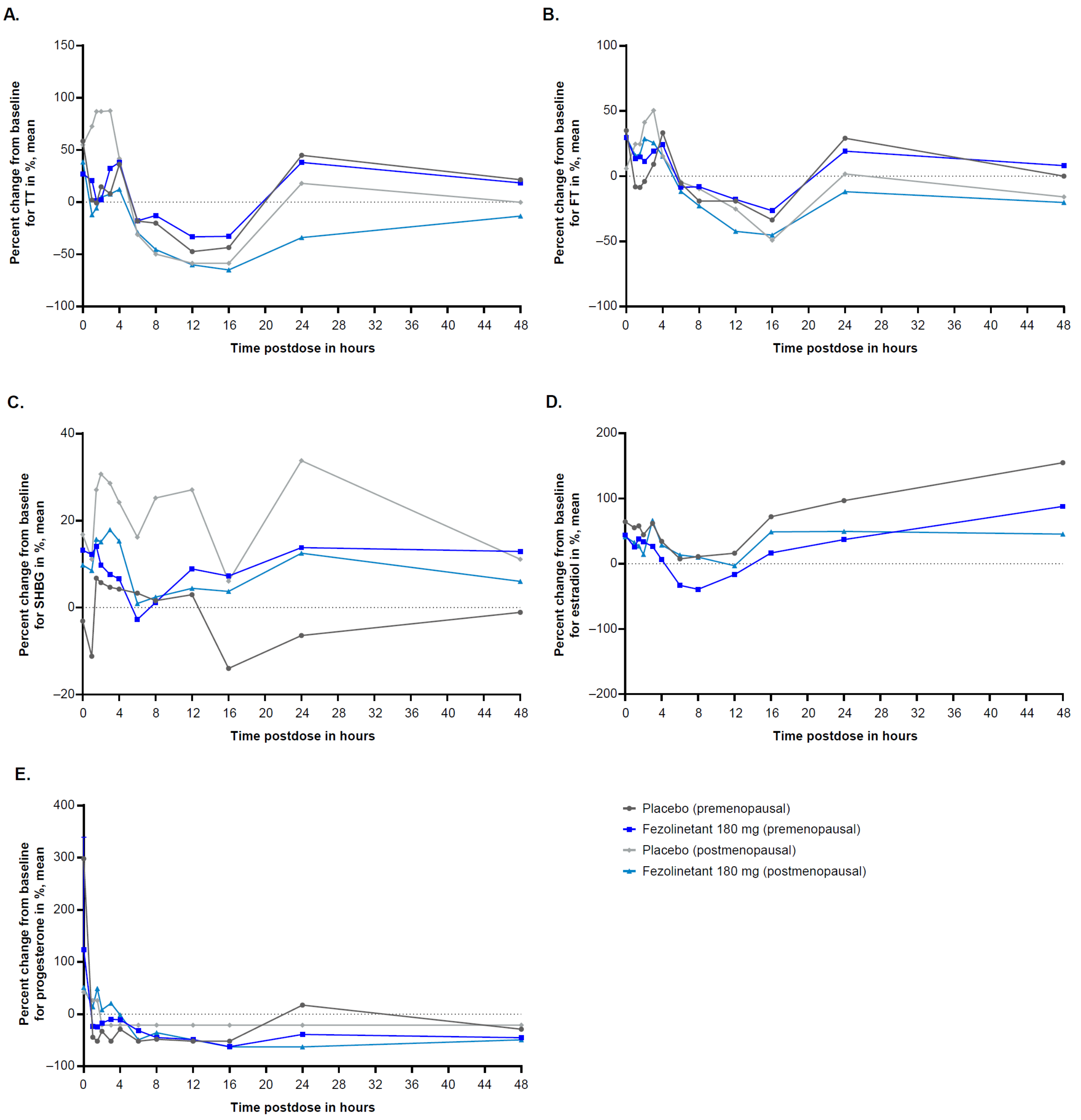

Numbers of participants – placebo (premenopausal; cohort 2-2a): two, fezolinetant 180 mg (premenopausal; cohort 2-2a): six (except for estradiol when n = 5 and progesterone when n = 4), placebo (postmenopausal; cohort 2-2b): two (except for estradiol when n = 0 and progesterone when n = 1), fezolinetant 180 mg (postmenopausal; cohort 2-2b): six (except for TT when n = 5, estradiol when n = 1, and progesterone when n = 2).

Baseline (time 0) is the mean pharmacodynamic data at three time points with at least two-hour intervals on Day −1.

FT, free testosterone; SD, standard deviation; SHBG, sex hormone binding globulin; TT, total testosterone.

Figure S6. Biomarker results in male participants (cohort 2-1) and premenopausal and postmenopausal female participants (cohorts 2-2a and 2-2b). Percent change in (A) TT, (B) FT, (C) SHBG, (D) estradiol, and (E) progesterone levels after multiple administrations of placebo or fezolinetant 180 mg (Day 10)

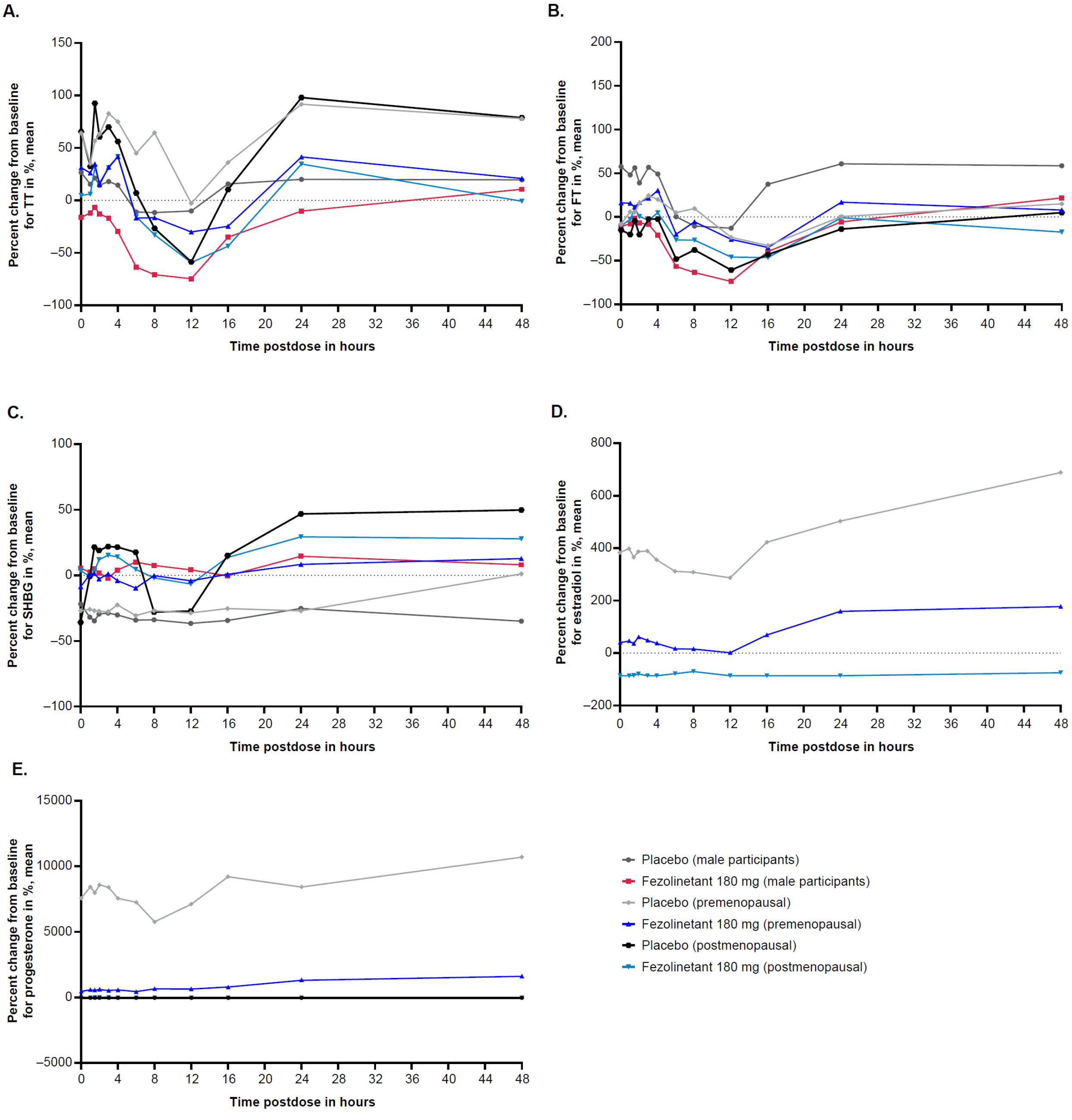

Serum samples from male participants were not evaluated for estradiol and progesterone concentrations.

Numbers of participants – placebo (male participants; cohort 2-1): three, fezolinetant 180 mg (male participants; cohort 2-1): eight, placebo (premenopausal; cohort 2-2a): two, fezolinetant 180 mg (premenopausal; cohort 2-2a): six (except for estradiol when n = 5 and progesterone when n = 4), placebo (postmenopausal; cohort 2-2b): two (except for estradiol when n = 0 and progesterone when n = 1), fezolinetant 180 mg (postmenopausal; cohort 2-2b): five (except for TT when n = 4 and estradiol and progesterone when n = 1).

Baseline (time 0) is the mean pharmacodynamic data at three time points with at least two-hour intervals on Day −1.

FT, free testosterone; SD, standard deviation; SHBG, sex hormone binding globulin; TT, total testosterone.
